# Influence of Co Doping on Copper Nanoclusters for CO_2_ Electroreduction

**DOI:** 10.1021/acsomega.4c07514

**Published:** 2024-11-11

**Authors:** Guilherme R. Nascimento, Marionir M. C. B. Neto, Juarez L. F. Da Silva, Breno R. L. Galvão

**Affiliations:** † Centro Federal de Educação Tecnológica de Minas Gerais, CEFET-MG, Av. Amazonas 5253, 30421-169 Belo Horizonte, Minas Gerais, Brazil; ‡ São Carlos Institute of Chemistry, University of São Paulo, P.O. Box 780, 13560-970 São Carlos, São Paulo, Brazil

## Abstract

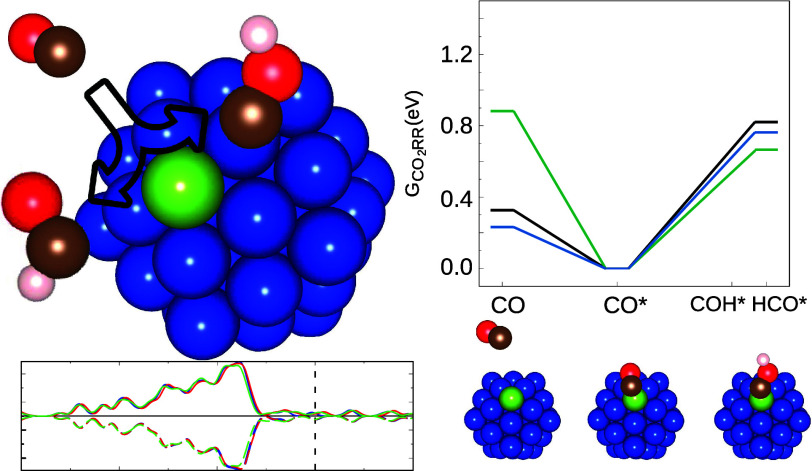

Copper stands out as one of the few metals capable of reducing carbon dioxide (CO_2_) beyond carbon monoxide (CO) and formic acid (HCOOH). Furthermore, substitutional doping in nanoclusters (NCs) has been expected to enhance their catalytic performance, even though our atomistic understanding of the influence of dopants is far from complete. Here, we investigate the effects induced by cobalt (Co) substitution doping in the Cu_55_ NC on the electroreduction of CO_2_ using density functional theory calculations combined with the computational hydrogen electrode model. We found that the replacement of a single copper atom in Cu_55_ by Co is energetically favorable, and it induces a drastic change in the density of states, for example, the appearance of a sharp peak near the Fermi level. The presence of a dopant atom on the surface increases the adsorption strength for all reaction intermediates, while also changing the preference of the adsorption site for selected species. The presence of the dopant atom on the surface of the particle hinders the production of CO in favor of more reduced products such as methane and methanol. From our analysis, it was observed that the catalyst will not suffer from poisoning by the OH species. However, our calculations predict that the catalysts will also enhance the formation of hydrogen in a competing reaction.

## 1 Introduction

The increase in carbon dioxide (CO_2_) emissions into the atmosphere, predominantly driven by human-induced activities, has caused significant environmental challenges, the greenhouse effect being central to these issues.^1,2^ CO_2_ is instrumental in disrupting the climatic equilibrium of the planet, thus affecting life globally. As a result, the pursuit of strategies to mitigate CO_2_ emissions has become urgent, a promising strategy being its conversion into valuable products.^1,3,4^ This approach presents an opportunity to alleviate environmental consequences while simultaneously contributing to the sustainable progression of our economy.

One of the most promising approaches for the conversion of CO_2_ is its electrochemical reduction,^5−7^ which can use renewable energy sources to convert CO_2_ into carbon monoxide (CO), ethylene (C_2_H_4_), various hydrocarbons and oxygenates.^6^ A key advantage of this approach is its scalability,^8^ however, it competes with the hydrogen evolution reaction (HER),^9,10^ which exhibits a more facile kinetics. Therefore, the development of catalysts that can improve the efficiency toward the desired product, while inhibiting HER, becomes essential.^11−14^

A deep understanding of the mechanisms and atomistic details is crucial, as it can directly contribute to the development of more effective catalytic systems. Several catalyst types have been experimentally tested^15−17^ and also computationally screened^18−23^ with the aim of reducing the required over potential. Among several potential catalysts, copper-based catalysts have the ability to generate alcohols and hydrocarbons from CO_2_, as well as variability in their selectivity and activity, which are intrinsically related to their structural properties.^9^ For an extensive overview of current advances in electrocatalysis that involve atomically precise nanoclusters, the reader is referred to the work of Zhao et al.,^24^ which also emphasizes the significance of computational predictions in improving our atomistic understanding of chemical reactions.

The incorporation of transition-metal dopants offers considerable promise for enhancing catalytic performance by altering the electronic structure, as has been demonstrated in recent studies.^25−28^ A mixture of copper (Cu) and another metal will affect the interaction of the different reactants, intermediates and products on the catalyst surface, allowing the materials to be fine-tuned to the desired catalytic properties.^29^ For example, the effect induced by the incorporation of cobalt (Co) into copper nanoparticles to their CO_2_RR performance^30^ has been experimentally investigated for several Co compositions. A small percentage of Co in the nanoparticle’s surface (3%, measured by X-ray photoelectron spectroscopy), has been found to yield a higher Faradaic efficiency in CO_2_RR and lower production of H_2_ compared to unary Cu nanoparticles of similar size. However, these improvements are lost for higher cobalt content. Similarly, it was found^30^ that the smallest particles (1.2 nm) were the most active. Although computational calculations have been performed, especially on metallic surfaces, we still lack a deep atomistic understanding of the role of single-atom-doped nanoclusters at nanometer sizes as catalysts for the CO_2_RR.

In light of the experimental verification of the significant influence exerted by the Co atom at minimal concentrations on finite-size particles,^30,31^ this investigation employs density functional theory (DFT) calculations alongside the computational hydrogen electrode (CHE) method to explore CO_2_RR in a Cu_55_ nanocluster and its Co-doped variant. The Cu_55_ nanocluster was selected as a model due to the high energy stability observed for several metals,^32^ which stems from the formation of a 55-atom Mackay icosahedron structure. Our findings indicate that the presence of the dopant atom on the surface of the particle reduces the generation of CO, while favoring the production of more reduced compounds such as methane and methanol. We also anticipate that the catalyst will be resistant to poisoning by OH. However, our computational results suggest that these catalysts may also promote the competing hydrogen evolution reaction.

## 2 Theoretical Approach and Computational Details

### 2.1 Total Energy Calculations

Our spin-polarized calculations are based on the DFT framework, as implemented in the Vienna *Ab initio* Simulation Package (VASP),^33,34^ version 5.4.4, which uses the projected augmented wave (PAW) method^34,35^ to describe the interaction between the core and valence electrons. The semilocal Perdew–Burke–Ernzerhof (PBE) formulation^36^ was used for the exchange-correlation energy functional, supplemented by the semiempirical D3 van der Waals (vdW) correction proposed by Grimme,^37^ which has been used to improve the description of the adsorption of chemical species.^38−42^

For all calculations, a cubic simulation cell was used, ensuring a minimum space separation of 15.0 Å in all directions between the particles and their periodic replicas, reducing their interactions, which is required to obtain accurate results for nonperiodic systems. Thus, it results in cubic boxes of 23 Å. Furthermore, because of the large size of the unit cell, there is no dispersion in the electronic states within the Brillouin zone (BZ), and hence only the Γ-point is used for the BZ integration.

To achieve our objectives, a systematic exploration of adsorption sites in undoped and doped Cu_55_ nanoclusters is required, significantly increasing computational cost. Consequently, to minimize computational cost, our methodology was divided into two distinct phases: (i) the preliminary exploration of the adsorption sites using cost-effective computational parameters (screening calculations) and (ii) the subsequent optimization of selected configurations using computationally larger parameters. For example, the following parameters were used:

- Screening adsorption site calculations: plane wave cutoff energy of 380.127 eV, which is 12.25% lower than the highest recommended plane wave cutoff energy (ENMAX_max_) among the selected species (Cu, Co O, C, H). A self-consistency convergence criterion of 1.0 × 10^–4^ eV was used for the total electronic energy and 0.10 eV Å^–1^ to obtain the equilibrium structures. The atomic positions of the nanocluster atoms were frozen in their gas phase positions, while only the atomic positions of the molecules were optimized.
- Final geometric optimizations: Plane wave cutoff energy of 488.735 eV, which is 12.25% higher than the ENMAX_max_ parameter. For the electron density self-consistency, we used 1.0 × 10^–5^ eV for the total energy, while the equilibrium configurations were obtained once the atomic forces on all atoms were smaller than 0.025 eV Å^–1^. All atoms in the unit cell were relaxed.

#### 2.1.1 Molecular Configurations

##### 2.1.1.1 Cu_55_ Nanocluster

The first step of this work consisted in determining the lowest-energy structure of the Cu_55_ nanocluster without adsorbed molecules. Thus, several previously reported geometries for 55-atom metal nanoclusters were used as structure candidates,^32^ which were optimized once again using the mentioned computational parameters. Among the optimized structures, we selected the Cu_55_ structure with the lowest energy for further calculations, namely (i) substitutional Co doping of the Cu_55_ nanocluster and (ii) adsorption of molecular species. For doping calculations, one Cu atom is replaced by one Co in each nonequivalent positions of the NC.

##### 2.1.1.2 Adsorbed Molecules

For studying the CO_2_RR over the optimized nanoclusters, we follow previous work^22^ and assess the suitability of each of them as active for CO_2_RR toward products more reduced than CO using four criteria based on information from the literature:^43−45^ (i) the free energy required for the hydrogenation of *CO into either *COH or *HCO must be small (as this is the potential determining step for the CO_2_RR to form products more reduced than CO); (ii) The CO adsorption energy has to be high enough to prevent *CO desorption; (iii) the OH bond to the nanocluster must be weak enough to avoid poisoning the catalyst with *OH; (iv) the free energy for the HER should not be too low, such as to make this competing reaction dominant.

**Figure 1:**
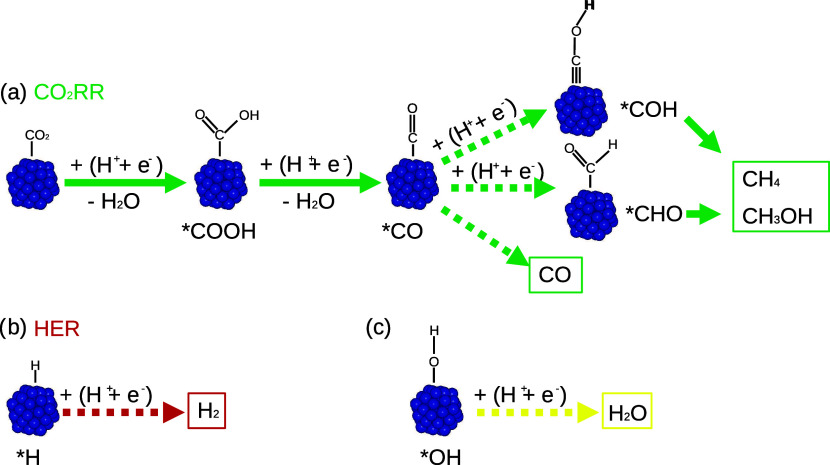
Schematic pathways of the reactions involved in the CO_2_ reduction process. (a) indicates the actual CO_2_RR steps, with dashed arrows highlighting those considered in this study. Similarly, (b) illustrates the competing hydrogen evolution reaction, while (c) shows the hydrogenation of OH, which is important to account for OH poisoning.

Figure 1 provides a simplified overview of the CO_2_RR pathways to CO, CH_4_ and methanol (CH_3_OH), together with competing HER and hydrogenation of OH on the surface. This figure highlights the explicit steps considered in this work to verify if the proposed materials meet the criteria described in the previous paragraph. To achieve the proposed goal, we explore the adsorption of H, OH, CO, COH, and HCO.

##### 2.1.1.3 Adsorption Structures

The first step was to perform a large exploration of possible adsorption configurations using the unary Cu_55_ with the calculation parameters at the screening level. For the H, OH, and CO species, we generate adsorption configurations taking into account half of the cluster (due to its symmetry). In total, 26 sites were proposed to start the calculations, which were 6 in the top position, 10 on the bridge, and 10 in hollow sites.

The adsorption of molecules such as COH and HCO on Cu_55_ can yield a large number of nonequivalent adsorption sites because of the possible orientations of the adsorbates, including monodentated and bidentated configurations. To sample the various possible adsorption configurations, we used an algorithm^46^ which operates as follows: (i) 100 million random adsorbed configurations are generated for the selected molecule in the NC using a cluster-molecule distance threshold to avoid too long/too short configurations. (ii) To reduce our structural database, similar or redundant structures are removed (as they can lead to the same local minimum structure upon geometry optimization). Through Euclidean distances (the distance between two coordinate vectors normalized by the sum of the module of the two vectors), it is possible to remove similar structures^46^ and we reduce the database to 5000 structures. Subsequently, these 5000 structures were further reduced to 50 using the *k-means* algorithm.^47^ This algorithm uses the eigenvalues of the Coulomb matrix as a representation for each structure,^48^ and uses them to divide the data set into 50 groups, from which the centroids are selected for DFT calculations.

### 2.2 Computational Hydrogen Electrode Model

The computational hydrogen electrode (CHE)^49^ is used to model the electrocatalytic steps of the reactions presented in Figure 1. At zero applied potential (*U* = 0 V vs RHE) and at any pH and temperature, the reaction
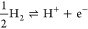
1is considered to be in equilibrium. This allows us to calculate the free energy of the electron–proton pair at any applied potential as *G*_H^+^+e^–^_ = *G*_H_2__ – eU.^49,50^

The Gibbs free energy at *T* = 298.15 K for each adsorbed molecule was calculated using the following equation:
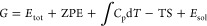
2where *E*_tot_ is the electronic total energy obtained by the DFT calculations. ZPE is the zero point energy, ∫*C*_p_d*T* the enthalpic contribution, – TS the entropic contribution and *E*_sol_ the correction of the solvation energy. Two sets of solvation energies were obtained from previous studies and are labeled SC1^50^ and SC2.^51^ The differences between these two distinct corrections are analyzed later.

All thermal corrections (ZPE, ∫*C*_p_d*T*, and TS) were obtained from the vibrational frequencies of the adsorbed molecules, calculated at the DFT level (the cluster was considered rigid in these calculations), and using the harmonic oscillator approximation and the thermochemistry module of the atomic simulation environment package.^52^ The following equations are used:
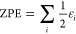
3
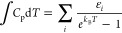
4
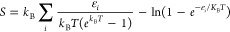
5where ε_*i*_ are the energies associated with the vibrational frequencies, *k*_B_ the Boltzmann constant, and *T* the temperature.

As in any computational prediction, deviations from the experiment can occur because of a suboptimal performance of the exchange-correlation functional, the omission of explicit water molecules, and others. However, it has been shown that the type of computational methodology employed here can predict onset potentials with a mean deviation from the experimental value of 0.07 V.^51^ Overall, this methodology offers sufficient predictive power to draw the conclusions that we aim at in this work.

## 3 Results and Discussion

### 3.1 Cu_55_ Nanocluster

**Figure 2:**
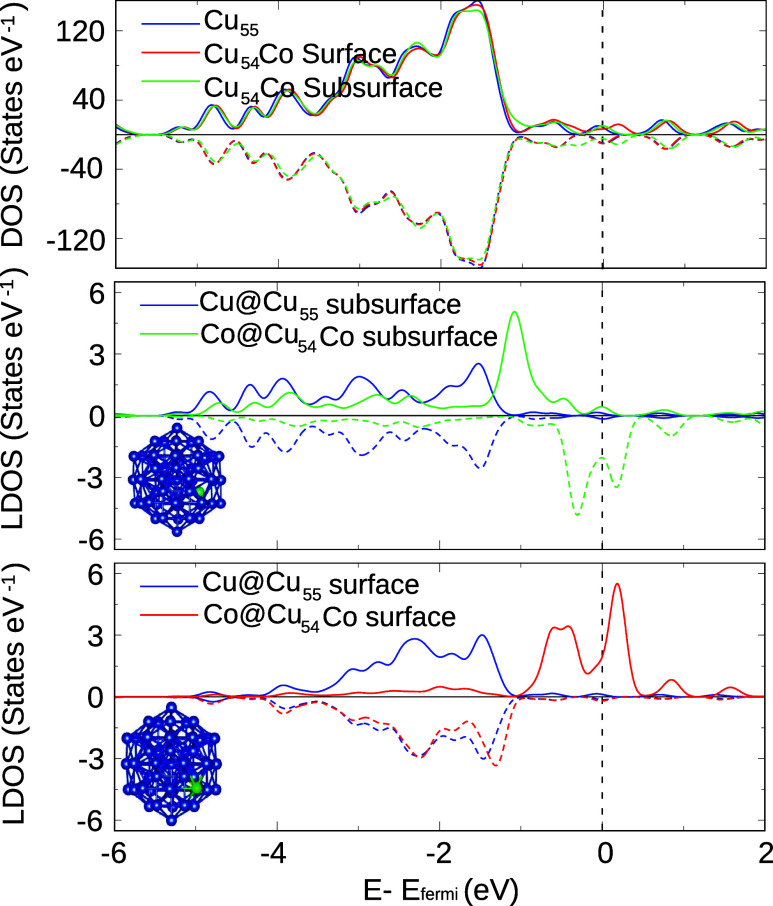
Density of states and Local density of states (d-states contributions) for unary and doped clusters. The top panel compares the total density of states of the substrates. The middle panel compares the contribution of a subsurface Co atom in Cu_54_Co with a subsurface Cu atom in Cu_55_. The lower panel shows a similar plot, but for the atoms on the surface. Continuous and dashed lines represent different spin contributions, while the vertical dashed line indicates the Fermi energy.

Upon conducting DFT optimizations on several model structures of Cu_55_ NC, the high-symmetry icosahedral (ICO) structure has been identified as the lowest-energy structure, which is consistent with previous DFT calculations.^32^ The ICO structure has one atom in the geometric center and 12 atoms in the first shell, while the remaining 42 atoms are located on the surface (second shell). Due to the high-symmetry of the ICO structure, there are only 5 nonequivalent atoms in the structure, which reduces the number of substitutional sites for the Co atoms to generate the Cu_54_Co NC, as shown in Figure S3 of the Supporting Information. We have performed DFT optimizations with the Co atom occupying each of them, and chosen the lowest-energy surface site and lowest-energy core site (subsurface) for the posterior analysis (see Figure S4 of the Supporting Information). Their structures are given in Figure 2.

The stabilities of the nondoped and doped NCs were assessed by calculating their binding energies per atom (*E*_b_), which are, respectively, written as
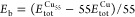
6and
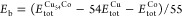
7In these equations, *E*_tot_^Cu_55_^ and *E*_tot_^Cu_54_Co^ are the total energies of the nondoped and doped NCs, while *E*_tot_^Cu^ and *E*_tot_^Co^ are the energies of the isolated atoms.

We have observed that the unary NC has a lower binding energy per atom (3.064 eV) than the doped NC (3.076 eV for the dopant on the surface and 3.084 eV for the dopant lying in the subsurface), meaning that the doped NC is more cohesively bonded than the doped one. To assess whether the replacement of a Cu atom of the unary copper NC by a Co one is a thermodynamically favorable process, we have calculated the energy for the reaction Cu_55_ + Co → Cu_54_Co + Cu using
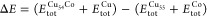
8The results show that this reaction is, in fact, exothermic, with Δ*E* of −1.11 and −0.64 eV for the dopant on the subsurface and surface, respectively.

To study the electronic structure of the atoms in the NC, we have also calculated their density of states (DOS), which is shown in Figure 2. The top panel provides the total DOS of the entire clusters, where the Co contribution is outweighed by the more numerous copper atoms. The graphs discriminate between the two spin components, and our calculations showed that all three NCs (unary and both doped cases) presented a magnetic moment of 3 μ_B_.

To understand the changes caused by the presence of the dopant, we compared the contribution of the cobalt atom at Cu_54_Co with that of a copper atom at Cu_55_ in an equivalent position (surface or subsurface). Through this figure, we can observe clear peaks near the Fermi level for the doped cases. This highlights the poor mixing in electronic density between the dopant and the rest of the cluster, which is characteristic of single-atom alloys,^53^ and are a possible reason for the high reactivity of such compounds.^53,54^

### 3.2 Analysis of the Adsorption Properties

After selecting unique and representative sites for each molecule in the Cu_55_ NC, we perform refinement calculations with the convergence parameters at the final level, which are then used to analyze the results. The adsorption of these molecules in the Co-doped clusters is performed at the final calculation level, starting from the optimized geometries of the unary NC as a reference, thus taking advantage of reasonable initial configurations. The reported adsorption energies (*E*_ad_) are at this final calculation level, and are obtained as
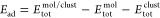
9where *E*_tot_^mol/clust^, *E*_tot_^mol^, and *E*_tot_^clust^ are, respectively, the total energy of the adsorbed configuration, isolated molecule and isolated cluster.

**Figure 3:**
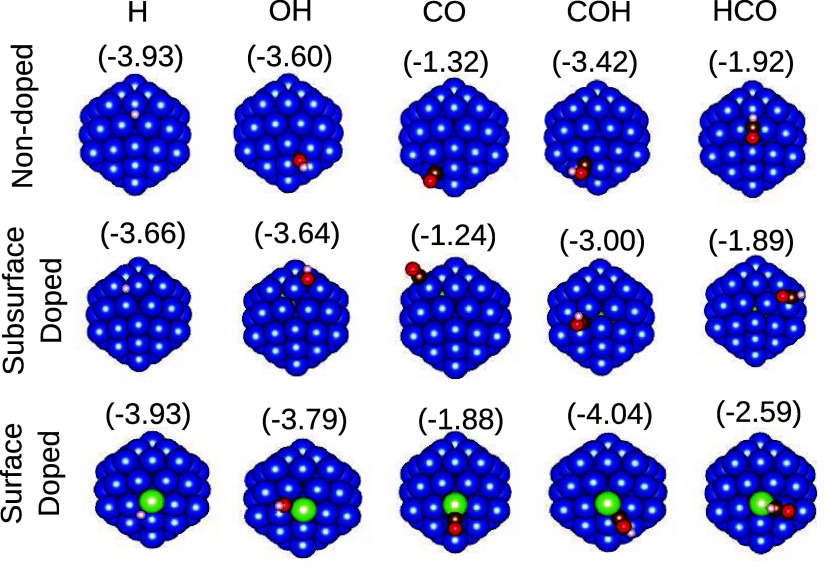
Most stable configurations for each adsorption systems. The values in parentheses are the adsorption energies in eV. Cu is shown in blue, Co in green, H in white, O in red, and C in brown.

Figure 3 and Table 1 provide details of the most stable structures obtained after exploring many adsorption sites (for results on all sites, see Supporting Information’s Section 4). It is seen that the hydrogen atom preferentially adsorbs on hollow sites on all three substrates. The OH radical also prefers hollow sites, except for the case with the dopant in the subsurface position, where it prefers to adsorb at a bridge site.

**Table 1:** Adsorption Energies and Structural Properties for the Most Stable Configuration for Each Molecule-Cluster Pair^a^

system	*E*_ad_ (eV)	*d*^TM–mol^ (Å)	*d*^OH^ (Å)	*d*^CO^ (Å)	θ (deg)
H@Cu55	–3.93	1.74			
H@Cu54Co surface	–3.93	1.74			
H@Cu54Co subsurface	–3.66	1.73			
OH@Cu55	–3.60	2.04	0.97		
OH@Cu54Co surface	–3.79	1.93	0.97		
OH@Cu54Co subsurface	–3.64	1.96	0.97		
CO@Cu55	–1.32	2.05		1.18	
CO@Cu54Co surface	–1.88	1.74		1.18	
CO@Cu54Co subsurface	–1.24	1.83		1.15	
COH@Cu55	–3.42	1.94	0.98	1.37	110
COH@Cu54Co surface	–4.04	1.77	0.98	1.35	110
COH@Cu54Co subsurface	–3.00	1.94	0.98	1.37	110
HCO@Cu55	–1.92	2.04		1.28	115
HCO@Cu54Co surface	–2.59	1.75		1.24	121
HCO@Cu54Co subsurface	–1.89	1.94		1.28	114

a The variable *d*^TM–mol^ represents the shortest distance between an atom in the molecule and an atom in the cluster, while *d*^OH^, *d*^CO^, and θ are distances and angles within the adsorbed molecule.

The CO molecule, the only closed shell intermediate considered here, adsorbs preferentially at a hollow site of the unary cluster (top and bridge local minima were also observed; see SI), whereas in both doped cases only top configurations are obtained as minima. Recall that the doped clusters have a very distinct density of states near the Fermi level, which may explain their distinct adsorption properties toward this stable molecule. For the two hydrogenated carbon monoxide isomers, COH adsorb mainly to hollow sites, with a few exceptions of high energy. HCO, which has the carbon atom bonded to hydrogen, oxygen, and a metallic atom, prefers to adsorb on the bridge sites, although we also observed the low-lying top and hollow sites (see SI).

Figure 3 also shows the adsorption energy for each system, where it can be seen that its magnitude for the surface-doped cluster is considerably higher than for the subsurface one, for all 5 species. This is consistent with the analyses of the thermodynamic and electronic stability characteristics between these two doping positions. It can be seen that all energies fall within the chemisorption range, although CO is less strongly bonded. It is also important to note that when comparing total energies alone, the HCO-Cu_55_ system is lower in energy than COH-Cu_55_, but this trend reverses for adsorption energies, because the isolated COH molecule is much less stable than HCO.

### 3.3 Computational Hydrogen Electrode Model

**Figure 4:**
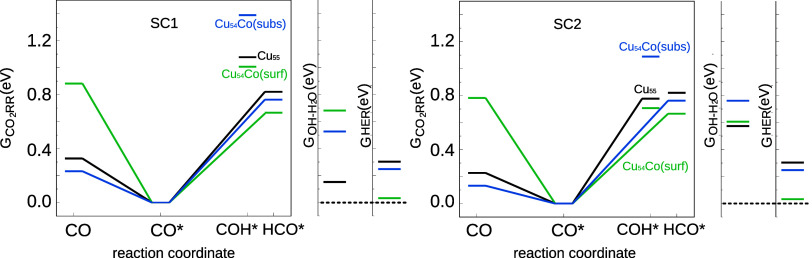
Free energy diagrams for selected processes involved in the electroreduction of CO_2_. For comparison, we show the results obtained with the solvation energies obtained through the two methods reported in this work (SC1 and SC2).

We conducted a harmonic vibrational analysis and calculated the Gibbs free energy in the minimum energy configurations presented in Figure 3. The values of each contribution to the free energy (ZPE, enthalpic, entropic, and solvation) are presented in Section 5 of the Supporting Information. The Gibbs free energy changes for the selected steps of CO_2_RR are provided in Figure 4. Recall that within the CHE model, the change in free energy from *CO to *COH/CHO can be calculated as . For comparison, Figure 4 is separated in two panels each showing the results of using a different solvation correction. This figure also incorporates side panels with Gibbs free energies of related processes (possibility of poisoning by adsorbed OH and HER).

Focusing first on the possibility of CO desorption, we see that the presence of the cobalt atom in the subsurface position leads to a small decrease in the desorption free energy, whereas if the dopant is in the surface position, a large increase is observed. Thus, we can already infer that an isolated Co atom on the surface of the nanoparticle will reduce faradaic efficiency for CO production. We now turn our attention to the possibility that the decrease in CO yield can be accompanied by an increase in the production of more reduced products (such as methane or methanol). For all such products, it is widely known that PDS resides in the hydrogenation of adsorbed carbon monoxide toward COH or HCO.

Figure 4 shows that the cobalt atom at the surface position indeed reduces the free energy required for this step compared to that of nondoped copper, while again the cobalt at the subsurface position is detrimental to reactivity. This holds true for both solvation corrections employed. It is seen that, for the doped clusters, HCO is preferred over COH for both solvation corrections, although in the SC2 case a much larger difference is observed.

However, calculations cannot focus only on the main CO_2_RR path, because HER is known to be a strong competitor in electroreduction. Furthermore, if the proposed material strongly binds OH, it is likely to be poisoned by this species. Starting with poisoning, it is seen in the side panels of Figure 4 that, although the doped nanoparticles increase the energy required for the OH removal step (compared to the nondoped case), this value still lies below the minimum energy required to proceed with CO_2_RR, and thus is not considered a problem. However, it turns out that the most promising catalysts so far (the nanoparticle with a single Co atom on the surface of the structure) largely facilitate the HER compared to the nondoped copper particle and thus will likely enhance the Faradaic efficiency of this competing step.

## 4 Conclusions

In this investigation, we presented a DFT study on the adsorption of key intermediates involved in the electrochemical reduction of CO_2_ (H, OH, CO, COH and HCO). The purpose of this study was to elucidate the underlying mechanisms of the process using undoped and Co-doped Cu_55_ nanoclusters. In addition, to design the nanocluster models, we performed a characterization of both undoped and Co-doped Cu_55_ nanoclusters. This characterization included the identification of the local minimum configurations for Cu_55_ and the determination of the optimal position of the substitutional Co dopant within the Cu_55_ nanocluster.

We have analyzed the thermodynamical stability of the doping process and the electronic structure of the three nanoparticles, where we found that the dopant promotes the appearance of new peaks in the density of states near the Fermi level. After performing a large number of geometry optimizations for all selected intermediates and clusters, we have discussed how the dopant atom changes the site preference of each molecule and found that the most affected species are CO and OH. The values of the adsorption energy for each species are also calculated.

On the basis of the results obtained from our investigation of the most stable configurations, we conducted calculations using the computational hydrogen electrode model (CHE) to assess the free energy changes associated with the various electrochemical steps. Focusing only on the steps directly involved in CO_2_RR, our analysis led us to the conclusion that the dopant atom on the surface of the particle will reduce the formation of CO while promoting the formation of more reduced products. Although we also predicted that this process will not be hindered by the poisoning of the active sites by OH species, our calculations indicate that Co-doped Cu_55_ nanoclusters will also greatly enhance the competing hydrogen evolution reaction.

## Supporting Information Available

Computational details; further results on unary and doped Cu_55_ nanoclusters; further results and graphs regarding adsorption properties; energetic contributions from vibrational calculations (PDF)
