# Supplementary material for: Influence of Co Doping on Copper Nanoclusters for CO2 Electroreduction
Source: ACS Omega. 2024 Nov 11;9(47):47114–21. doi: 10.1021/acsomega.4c07514 (PMC11603234; doi:10.1021/acsomega.4c07514)
Supplement: Supplementary file 1 — ao4c07514_si_001.pdf [file ao4c07514_si_001.pdf]

Electronic Supporting Information: The Influence of Co Doping on Copper Nanoclusters for CO_2 Electroreduction

Guilherme R. Nascimento,[†] Marionir M. C. B. Neto,[†] Juarez L. F. Da Silva,[‡] and Breno R. L. Galvão[*,†]

†*Centro Federal de Educação Tecnológica de Minas Gerais, CEFET-MG, 30421-169, Belo Horizonte, MG, Brazil*

‡*São Carlos Institute of Chemistry, University of São Paulo, PO Box* 780, 13560-970, *São Carlos, SP, Brazil*

E-mail: brenogalvao@gmail.com

Contents

1 Introduction **S-2**

2 Theoretical Approach and Computational Details **S-2**

2.1 PAW Projectors: Technical Details S-2

2.2 Physical-chemical Properties S-3

2.2.1 Effective Coordination Number (*ECN*) S-3

3 Unary and Dopped Cu_{55} Nanoclusters **S-4**

3.1 Lowest Energy configurations for the unary cluster S-4

3.2 Cobalt dopant on Cu_{55} Nanoclusters S-5

4 **Adsorption Properties on Unary and Dopped Cu_{55} Nanoclusters** **S-7**

4.1 Results of the Screening Calculations S-7

4.1.1 H Adsorption S-7

4.1.2 OH Adsorption S-9

4.1.3 CO Adsorption S-11

4.1.4 COH Adsorption S-13

4.1.5 HCO Adsorption S-15

4.2 Results of the Refined Calculations S-16

4.2.1 H Adsorption S-17

4.2.2 OH Adsorption S-20

4.2.3 CO Adsorption S-23

4.2.4 COH Adsorption S-26

4.2.5 HCO Adsorption S-29

5 **Energetic Contributions from Vibrational Calculations** **S-32**

References **S-34**

1 Introduction

This document contains technical details and analyses that complement and support the main manuscript, such as PAW projector details, plane-wave cutoff energies, computational procedures and results that do not fit in the main manuscript.

2 Theoretical Approach and Computational Details

2.1 PAW Projectors: Technical Details

All calculations performed in this work employed the Vienna Ab initio Simulation Package (VASP). Table S1 shows the details of the Projector Augmented Wave (PAW) projectors used in `POTCAR` files and their versions. `ENMAX` specifies the recommended plane-wave cutoff

energy in eV and Z_{val} provides the number of valence electrons on each element. The PBE exchange-correlation functional[1] was employed.

Table S1: Technical details of the PAW-PBE projectors selected for this study. Recommended cutoff energy for the plane-wave basis set (ENMAX), number of valence electrons, (Z_{val}), and valence electronic configuration.

Element	PAW projector	ENMAX (eV)	Z_{val}	Valence
O	O_GW 19Mar2012	434.431	6	$2s^2 2p^4$
Cu	Cu_GW_new 19Mar2012	417.039	11	$3d^{10} 4s^1$
C	C_GW_new 19Mar2012	413.992	4	$2s^2 2p^2$
Co	Co_GW 31Mar2010	323.400	9	$3d^7 4s^2$
H	H_GW 21Apr2008	300.000	1	$1s^1$

2.2 Physical-chemical Properties

2.2.1 Effective Coordination Number (*ECN*)

ECN is an useful generalization of the classical definition of coordination number (number of nearest neighbors of an atom). In this approach, a different weight is calculated for each bond length with distace d_{ij}.[2] This approach is based on the fact that a particular atom i binds stronger with the closer j atoms, and hence, small changes in the coordination environments can be taken into account[3]. The ECN_i is obtained by the following set of equations:

$$ECN_i = \sum_j \exp\left[1 - \left(\frac{d_{ij}}{d^i_{av}}\right)^6\right] \tag{1}$$

where d_{ij} is the distance between atom i and j, while d^i_{av} is defined as:

$$d^i_{av} = \frac{\sum\limits_j d_{ij} \exp\left[1 - \left(\frac{d_{ij}}{d^i_{av}}\right)^6\right]}{\sum\limits_j \exp\left[1 - \left(\frac{d_{ij}}{d^i_{av}}\right)^6\right]} \tag{2}$$

The average *ECN* for a given structure can be obtained by

$$ECN = \frac{1}{N} \sum_{i=1}^{\mathrm{N}} ECN_i \tag{3}$$

where N is the number of atoms.

3 Unary and Dopped Cu_{55} Nanoclusters

3.1 Lowest Energy configurations for the unary cluster

The first step of this work consisted in the optimization of different geometries of Cu_{55} unary cluster using common structures found for clusters of 55 atoms:[4] cuboctahedron (CUB), distorderted reduced-core (DRC1 e DRC2), fragment face-centered cubic (FCCf), icosahedral (ICO), LOW, LOW-Au, tetrahedral-like (THL) and hexagonal close-packed wheel-type (WHE), as seen in Figure S1.

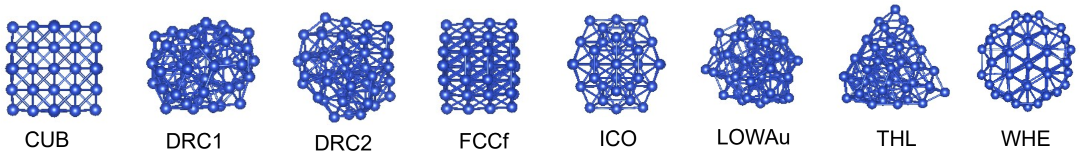

Figure S1: Structures obtained after geometry optimization for the Cu_{55} cluster.

The PBE functional does not account for dispersion forces, and therefore cannot be employed reliably on systems where the long-range interactions play an important role. To assess the impact of such forces, two calculations were performed to compare the energy values, one using the DFT-D3 correction method[5] and the other not. Figure S2 shows the results of the relative energies. For both results, the icosahedron (ICO) configuration presents the lowest energy, which is in agreement with previous results.[4] This figure also shows that the inclusion of dispersion corrections increases the energy difference between any structure and the global minimum.

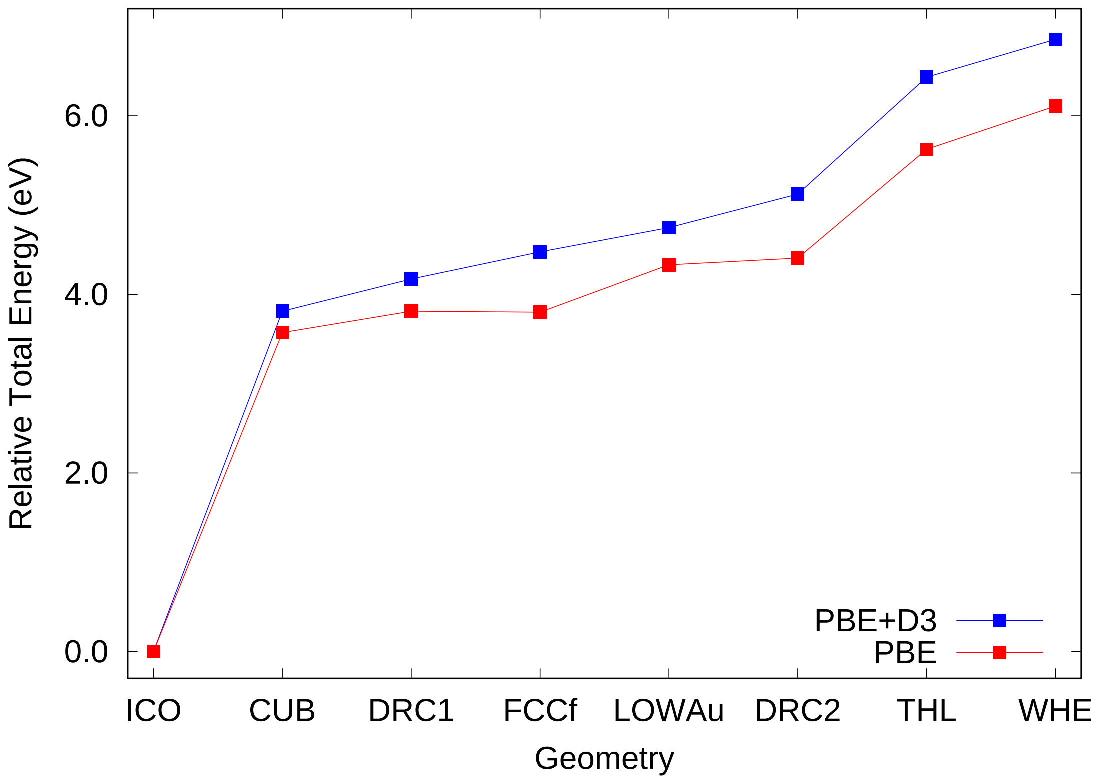

Figure S2: Relative energies for different model structures including dispersion corrections (PBE+D3) and without its inclusion (PBE).

Figure S1 showed the configurations obtained after the optimization. The icosahedral structure, which is the most stable configuration has one atom in the geometric center and twelve encased in the core. The remaining forty-two atoms are located on the surface. Thus, the icosahedral geometry of unary copper was selected as the most stable structure and therefore used in the other calculations of this work. The dispersion correction is also included in the calculations.

3.2 Cobalt dopant on Cu_{55} Nanoclusters

The icosahedron structure has five nonequivalent atomic sites which can be replaced by the cobalt dopant, as shown in Figure S3. In the core of the cluster there are 12 atoms in the M2 position with $ECN = 12.21$ and one atom in the geometric center with $ECN = 13.04$. In the surface, there are 23 atoms in the M3 position with $ECN = 7.90$, 6 atoms in the M5 position with a similar ECN, and 12 atoms at the M4 position with $ECN = 5.58$.

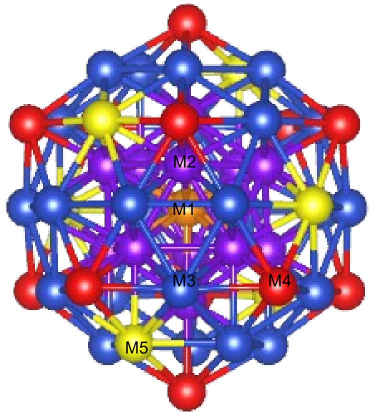

Figure S3: Icosahedron structure with 55 atoms. The 5 possible dopant sites are indicated with different colors.

The relative energy of the doped clusters with the cobalt in each position is shown in Figure S4, ordered by the *ECN* of the site. The results show that the lowest energy site is M2 (subsurface). For the calculations with the molecules adsorbed, we will use both the minimum energy structure (Co at M2 position), as well as the dopant on M4 in order to understand the effect of the dopant atom in the surface (note that this is lower in energy than the M3 and M5 surface positions).

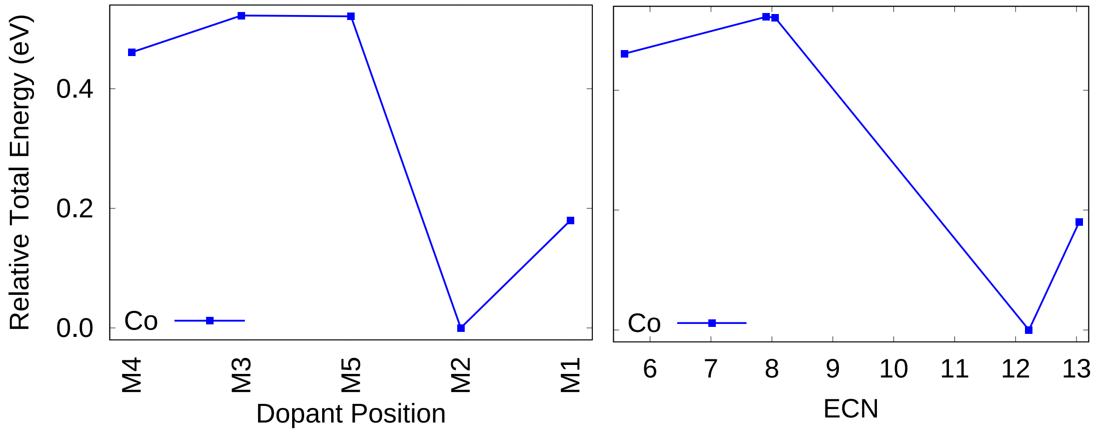

Figure S4: Relative energies for different dopant positions. Left panel gives the name of the dopant position as the x-axis, while the right one shows the *ECN*

4 Adsorption Properties on Unary and Dopped Cu_{55} Nanoclusters

As discussed in the main document, in this work we have performed a vast and preliminary exploration of the possible adsorption sites with reduced accuracy and computational cost (here termed as screening calculations) which was followed by a selected number of calculations using a more accurate level (termed final calculations). In this section, we start with the screening results and selection of sites, followed by the results of the final calculations. The several adsorption sites are classified between top (T): with the molecule above an atom of the cluster, bridge (B): above the middle point of 2 atoms, and hollow (H): above the middle point of 3 atoms. In naming the adsorption sites, the T, B and H indexes are followed by a number indicating the several geometry optimizations performed (*e.g.* T1, T2, B1, B2, H1, and so on).

4.1 Results of the Screening Calculations

4.1.1 H Adsorption

As can be seen in Figure S5, for the H atom adsorption the hollow sites present the lowest energies, while the top ones present the highest. It can also be noted the presence of five energy plateaus, where the lowest energies are represented by the H8, B5, B2, T1 and T2 sites respectively. These structures are shown in Figure S6 and are selected for the final calculations.

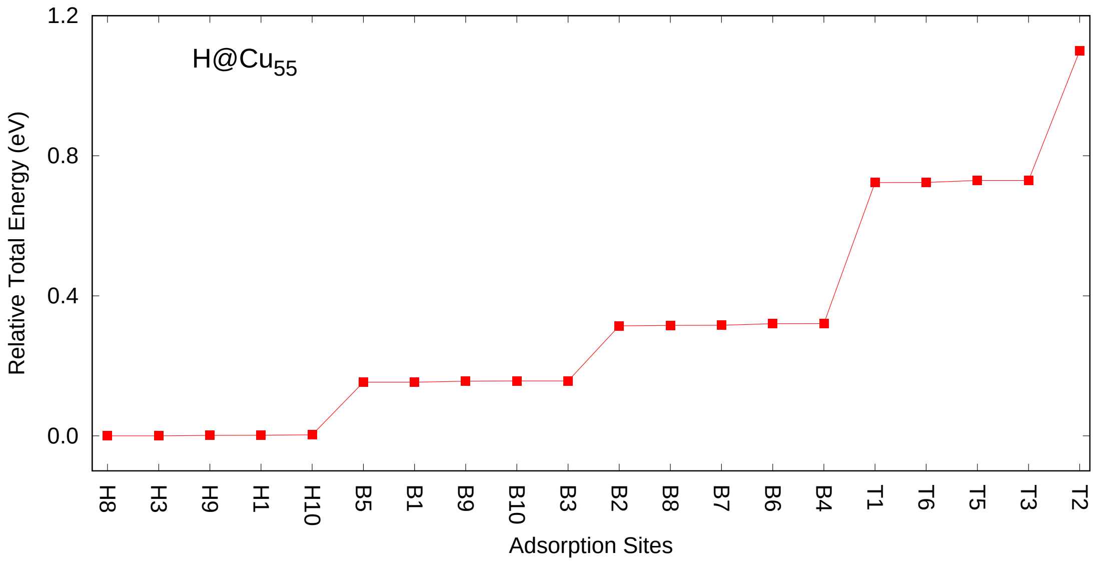

Figure S5: Relative energies of the optimized H adsorption configurations on the Cu_{55} cluster.

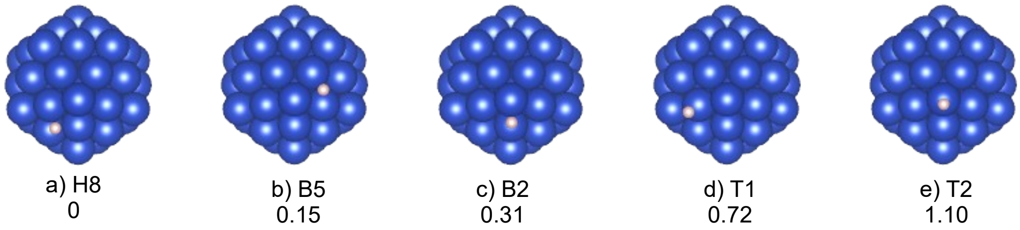

Figure S6: Configurations selected in the screening stage for H adsorption on Cu_{55} clusters with relative energies in eV. The atoms in blue indicate Cu and the atom in white indicates H.

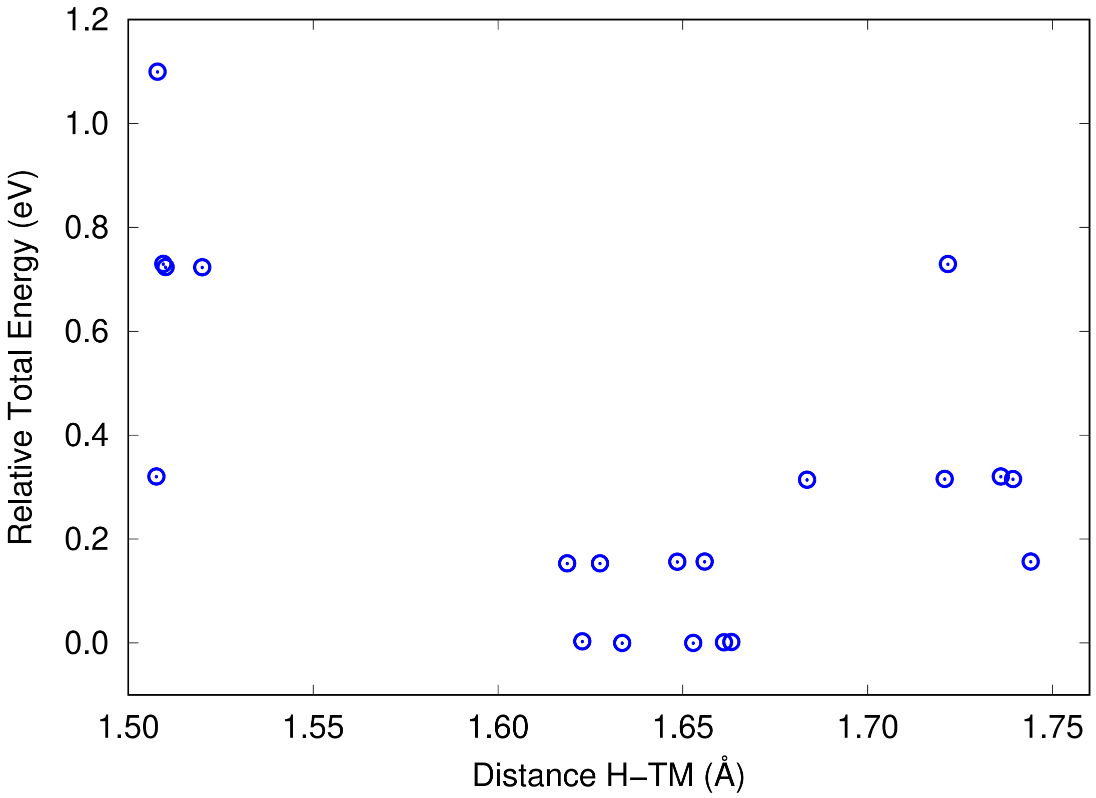

Figure S7: Relative energies of the adsorbed configurations of an H molecule over the Cu_{55} cluster as a function of the shortest Cu–H distance.

4.1.2 OH Adsorption

For the OH adsorption (Figure S8), there is no clear preference between hollow or bridge sites. Top sites tend to show higher energies. The oxygen ends the optimization bound to the cluster, even in optimizations where the H atom initiates closer to it. In this case, we chose the bridge and hollow sites of lower energies in each plateau, and two representative top sites, totalling eight structures for the final optimizations. These adsorption configurations are shown in Figure S9.

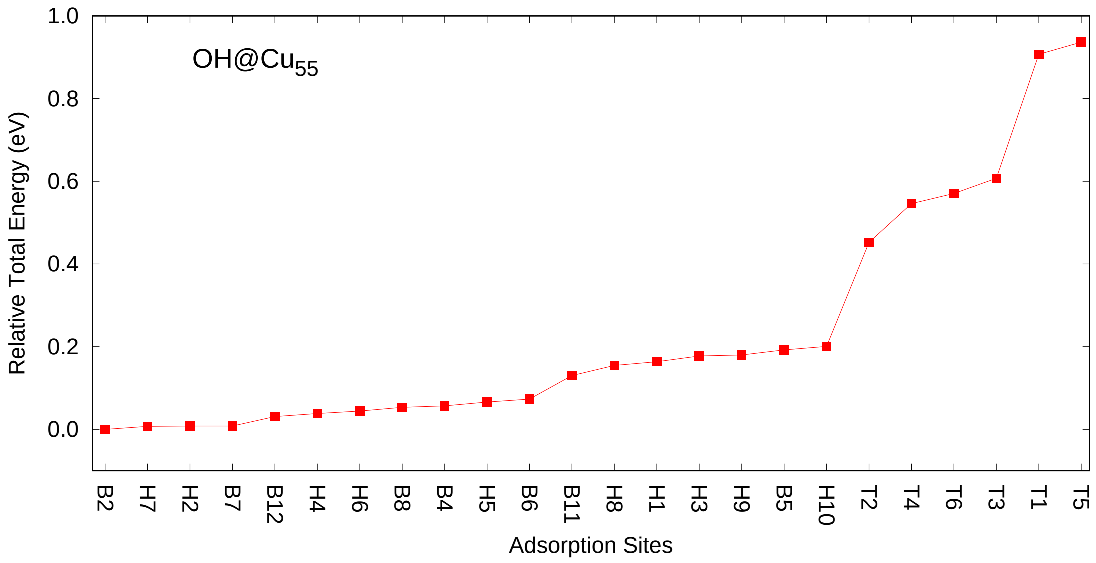

Figure S8: Relative energies of the optimized OH adsorption configurations on the Cu_{55} cluster.

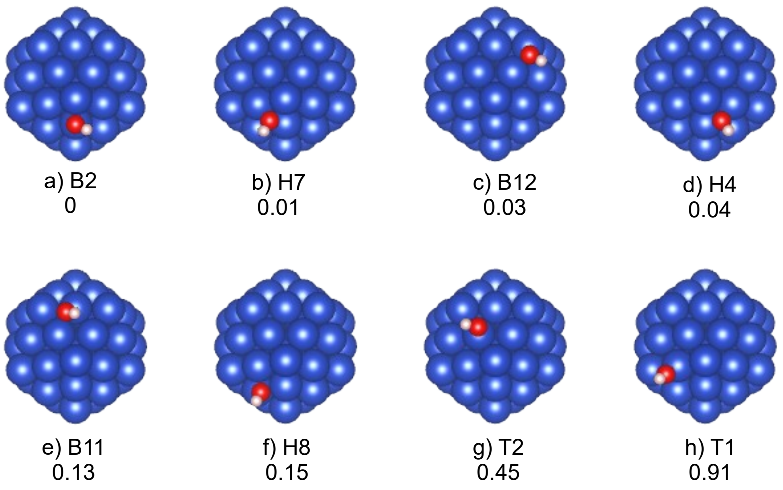

Figure S9: Configurations selected in the screening stage for OH adsorption on Cu_{55} system and relative energies in eV. Atoms in blue indicate Cu, while red and white indicate O and H respectively.

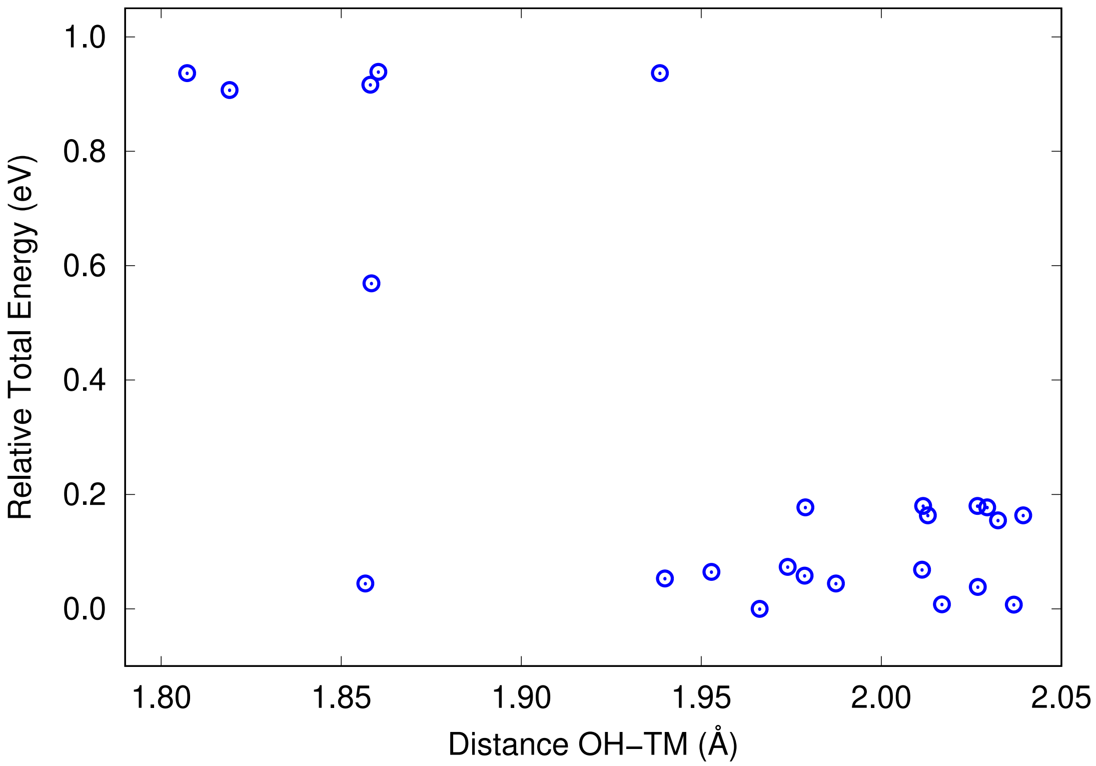

Figure S10: Relative energies of the adsorbed configurations of an OH molecule over the Cu_{55} cluster as a function of the shortest Cu – O distance.

4.1.3 CO Adsorption

According to Figure S11, we can conclude that for CO adsorption, the lowest energies are predominant in hollow sites, followed by bridge and top sites. However, an exception is seen for the B6 site, which shows the highest energy. In all systems, the C atom ends bonded to the cluster. For the CO adsorption, eight structures were chosen to perform the final optimization following a criterion similar to that of OH. These configurations are shown in Figure S12.

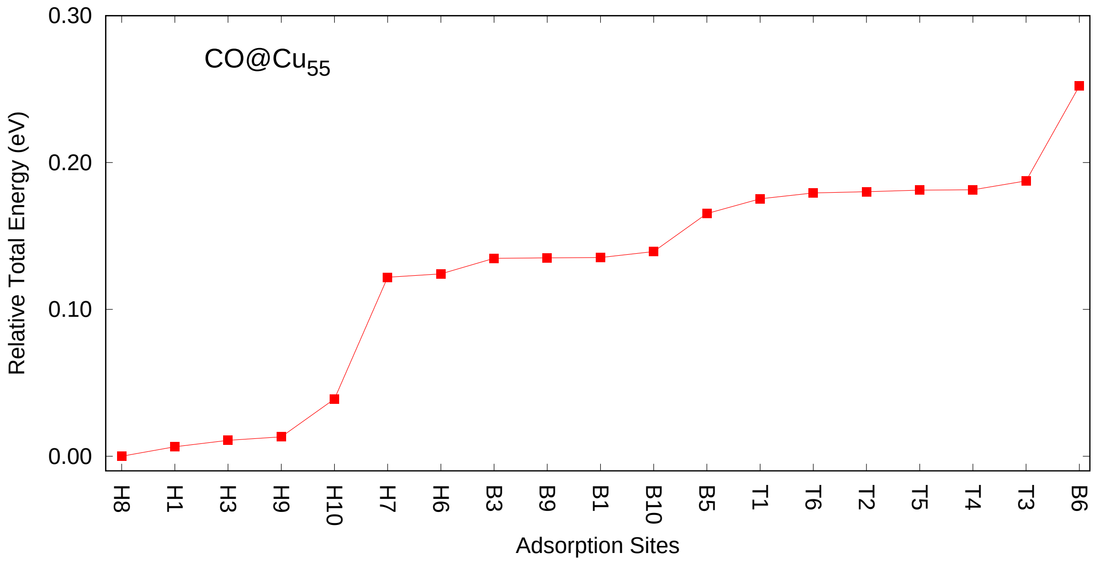

Figure S11: Relative energies of the optimized CO adsorption configurations on the Cu_{55} cluster.

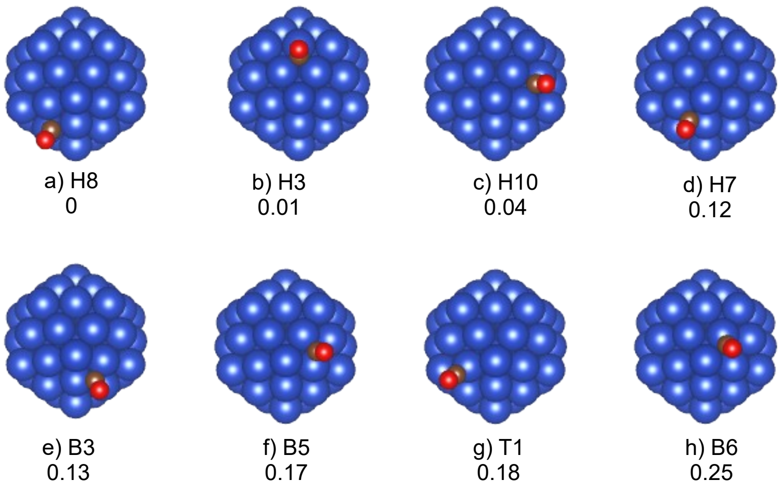

Figure S12: Configurations selected in the screening stage for CO adsorption on Cu_{55} system and relative energies in eV. The atoms in blue indicate Cu, while brown and red indicate C and O respectively.

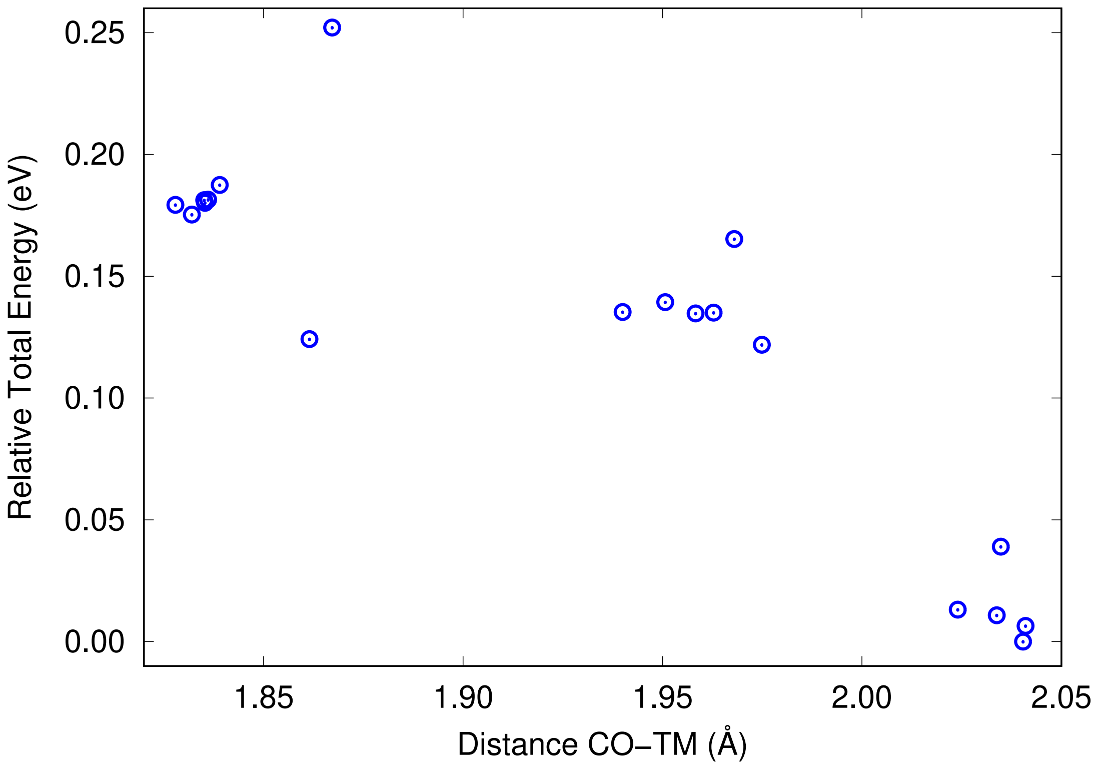

Figure S13: Relative energies of the adsorbed configurations of an CO molecule over the Cu_{55} cluster as function of the shortest Cu – C distance.

4.1.4 COH Adsorption

For the COH adsorption, and according to Figure S14, it is possible to conclude that the lowest energies are predominant in the hollow sites, i.e, above the middle point of 3 atoms. The higher energies are in the top sites, and no bridge site was achieved. In all systems, the carbon atom ends are bonded to the cluster.

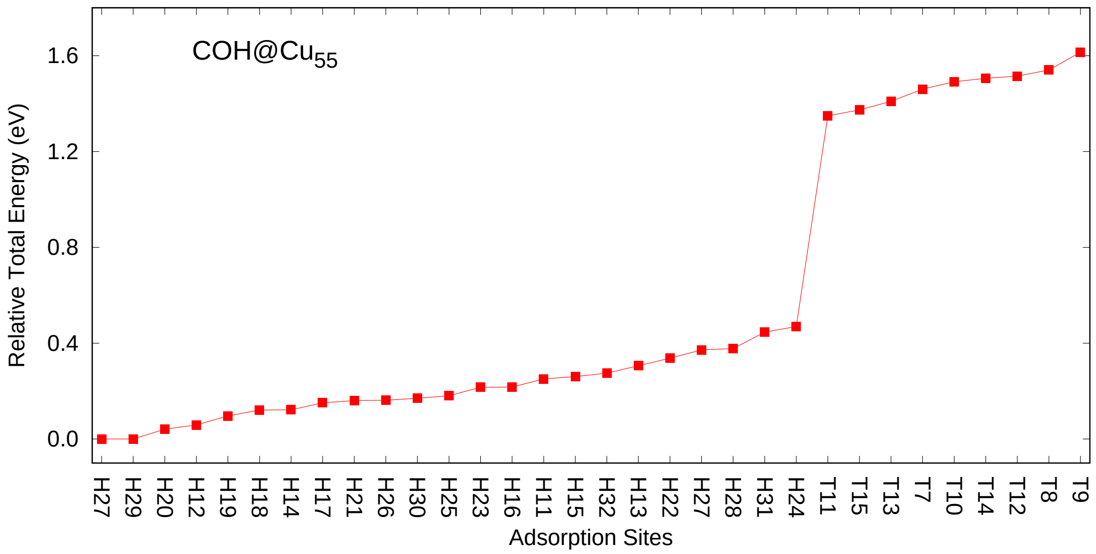

Figure S14: Relative energies of the optimized COH adsorption configurations on the Cu_{55} cluster.

For the COH adsorption, ten configurations were chosen to perform the final optimizations, which are given in Figure S14. They were chosen such as to ensure diversity (by removing similar structures or energies) and providing low energy candidates.

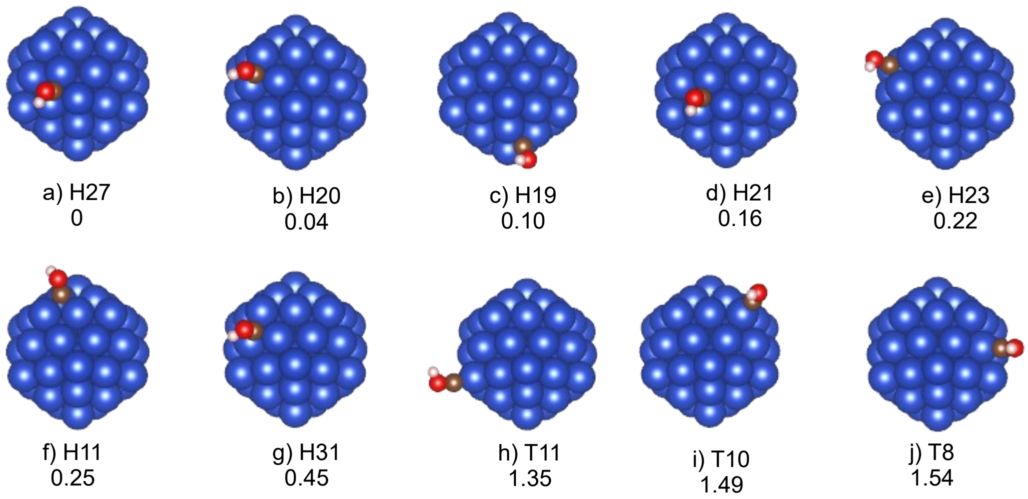

Figure S15: Configurations selected in the screening stage for COH and Cu_{55} system and relative energies eV. The atoms in blue indicate Cu and the atoms in brown, red, and white indicate C, O, and H respectively.

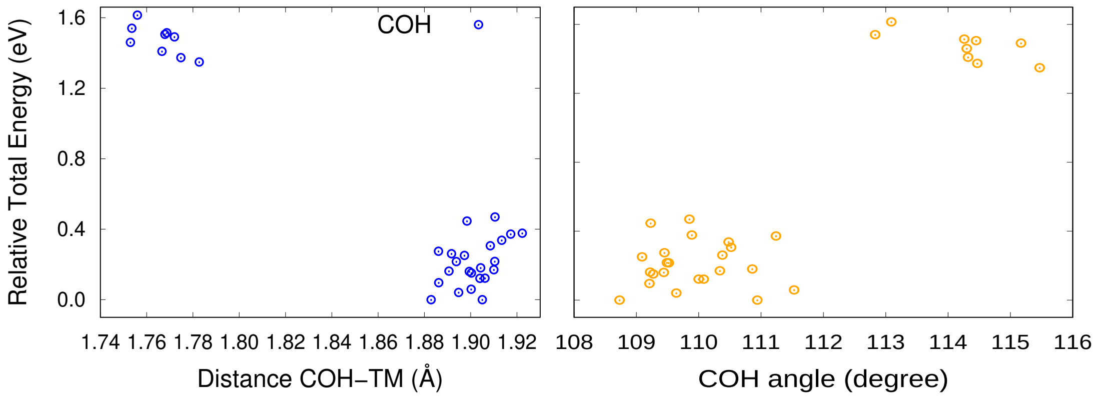

Figure S16: Relative energies of COH adsorbed on the Cu_{55} cluster as a function of the shortest Cu – C distance, and as a function of the angle formed by C, O and H atoms.

4.1.5 HCO Adsorption

The results for the HCO molecule are presented in Figure S17. In this case, several structures ended the optimization with a bidentate geometry (two atoms of the molecule are bonded to the cluster). The symbol * in the site label indicates that both the C and O atoms are bound to the cluster, while the symbol + indicates that the C and H atom are bonded. As seen in this figure, the B8* and B5* sites feature the lowest energies and the top sites feature higher energies. In most cases, the C atom is bonded to the cluster, except in T13 and T16, where the O ends are bonded to the cluster.

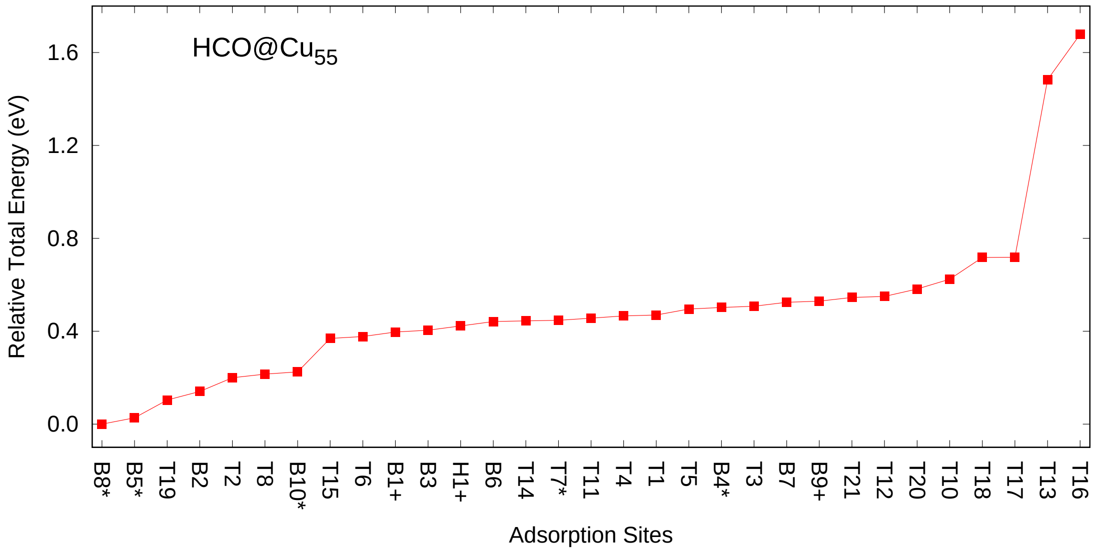

Figure S17: Relative energies of the optimized HCO adsorption configurations on the Cu_{55} cluster.

For the HCO adsorption, ten structures were chosen to perform the final optimizations.

These include bridge and hollow sites with bidentate geometry. The choice of representative structures of this system took into account the different structural models after the optimization, trying to select the structures with the lowest energies of the plateaus of the graph on Figure S17. The structures chosen to perform the final optimization are shown in Figure S18.

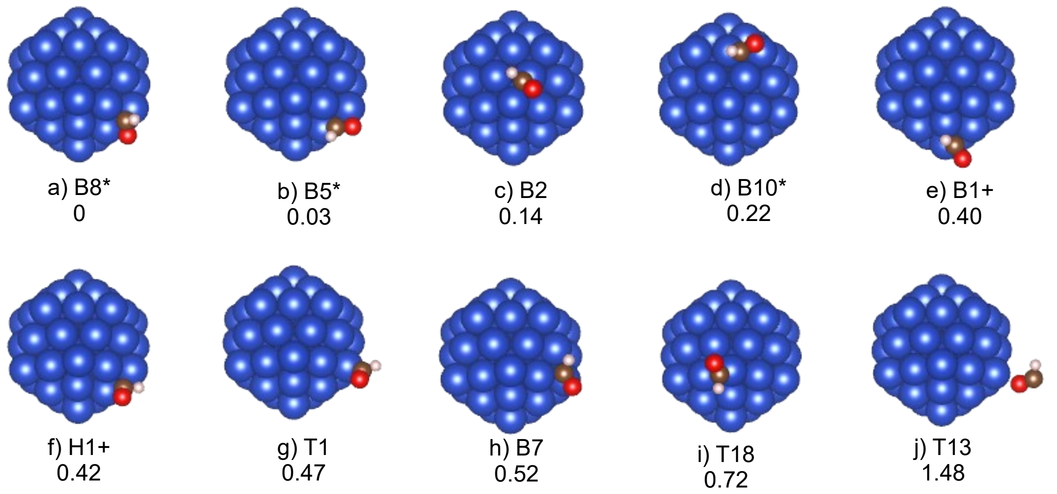

Figure S18: Configurations selected in the screening stage for HCO and Cu_{55} system and relative energies eV. The atoms in blue indicate Cu and the atoms in brown, red and white indicate C, O and H respectively.

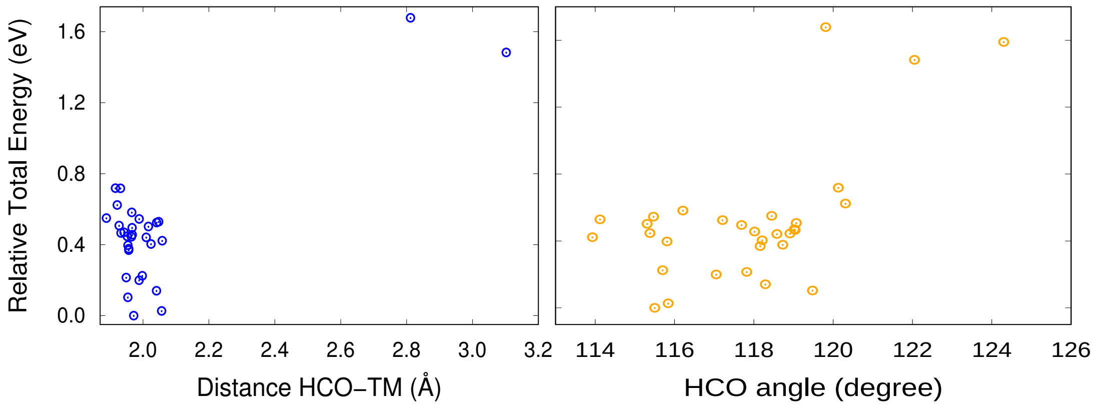

Figure S19: Relative energies of HCO adsorbed on the Cu_{55} cluster as a function of the shortest Cu–C distance, and as a function of the angle formed by H, C and O atoms.

4.2 Results of the Refined Calculations

The refined calculations on the structures selected in the previous section are now presented. The results of the adsorption when the cluster is non-doped and cobalt-doped (surface and subsurface) will be given.

4.2.1 H Adsorption

Figure S20 shows the relative energies for the H atom adsorption on the unary and doped clusters, while Figures S21, S22 and S23 show the adsorption sites, geometries, and relative energies after optimization on the three studied substrates. As seen, hollow sites are preferred for both for doped and non-doped clusters.

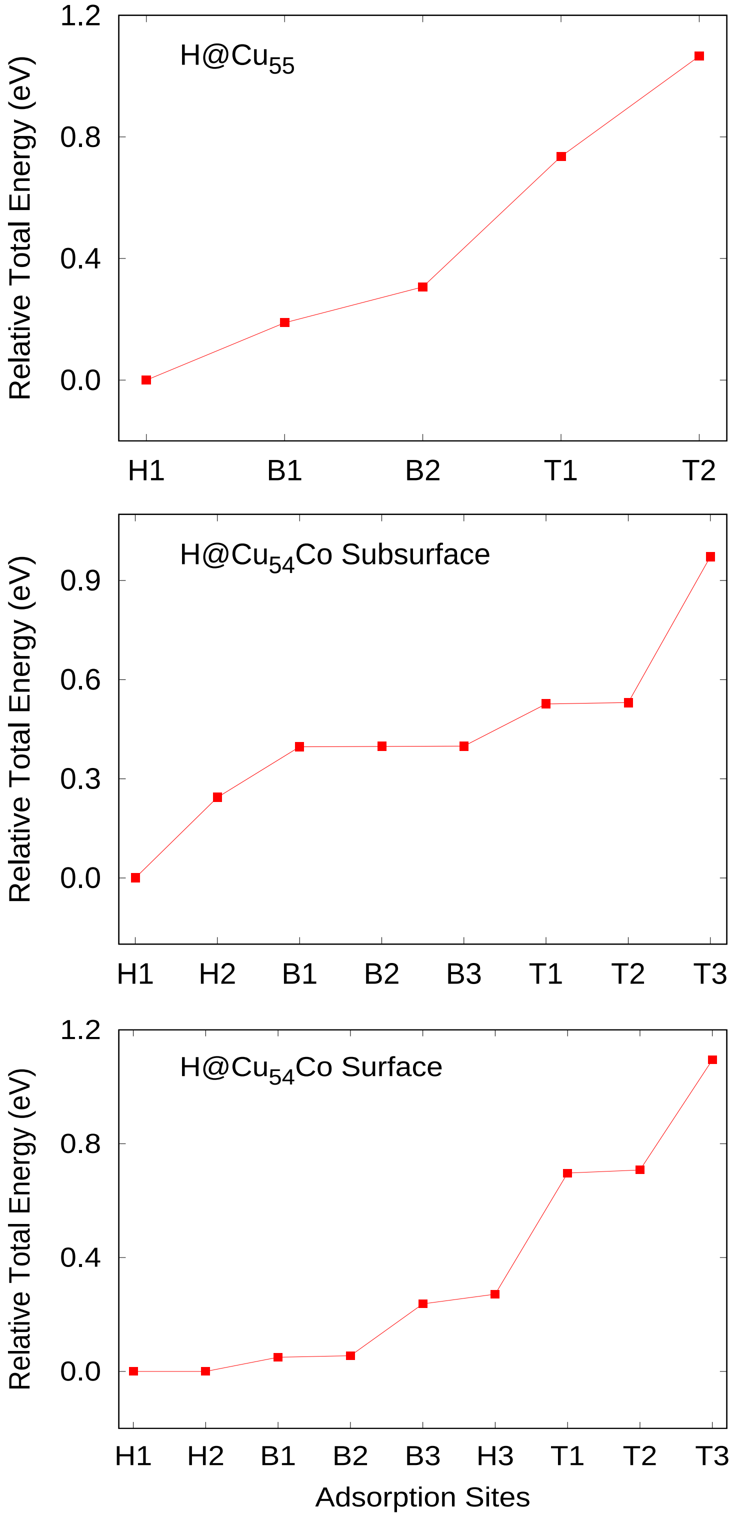

Figure S20: Relative energies of the optimized H adsorption configurations on the Cu_{55} and $Cu_{54}Co$ clusters.

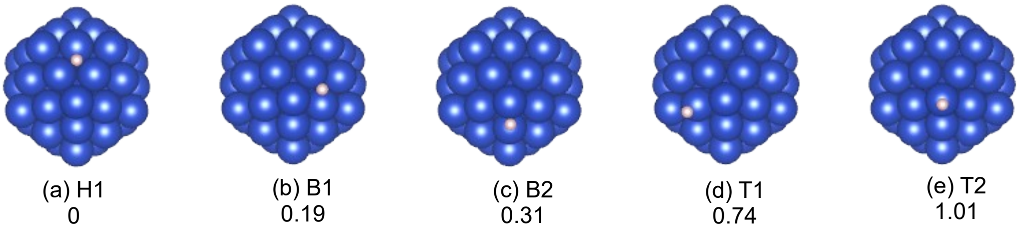

Figure S21: Optimized H@Cu_{55} configurations. Relative energies are given in eV.

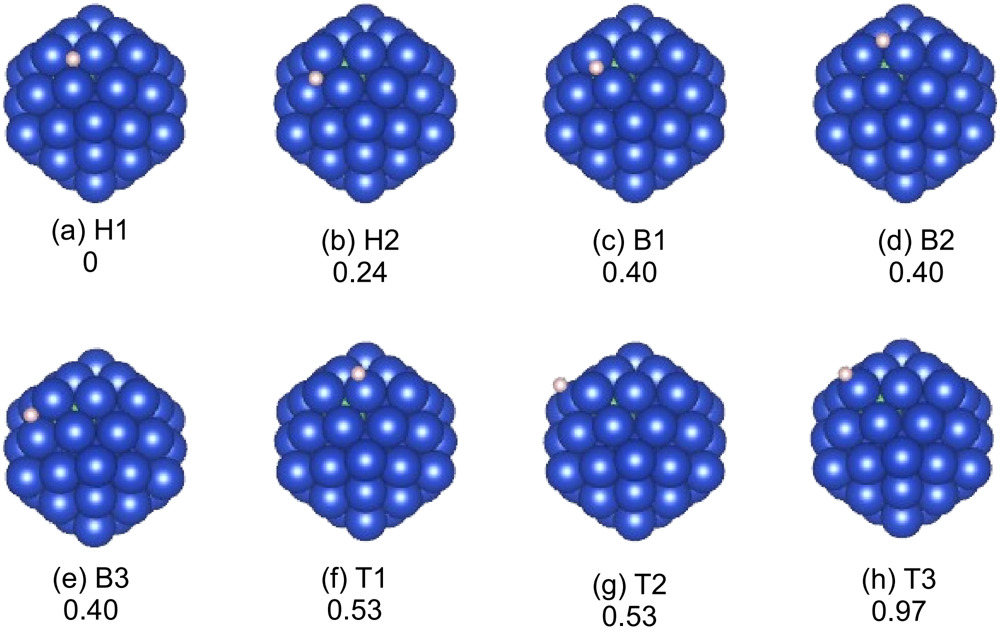

Figure S22: Optimized H@$Cu_{54}Co$ configurations with the dopant in the subsurface position. Relative energies are given in eV.

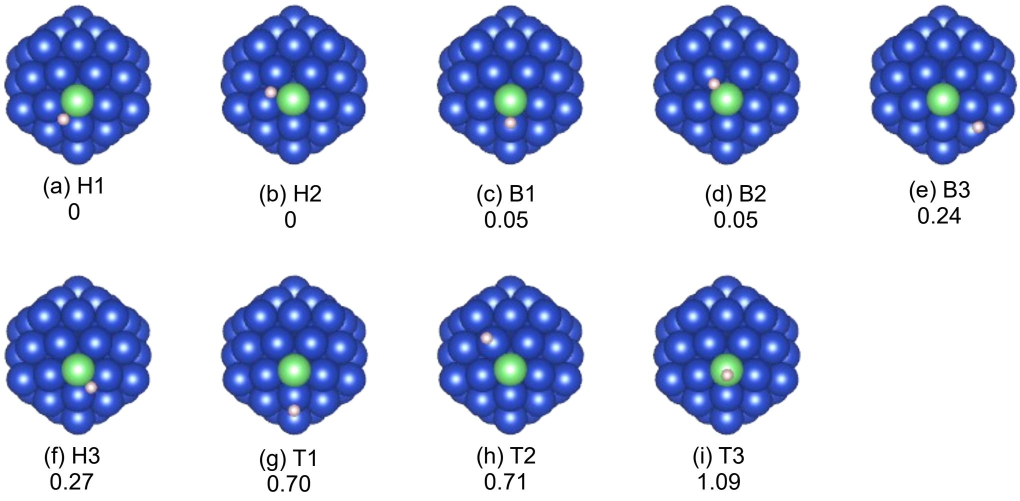

Figure S23: Optimized H@Cu_{54}Co configurations with the dopant in the surface position. Relative energies are given in eV.

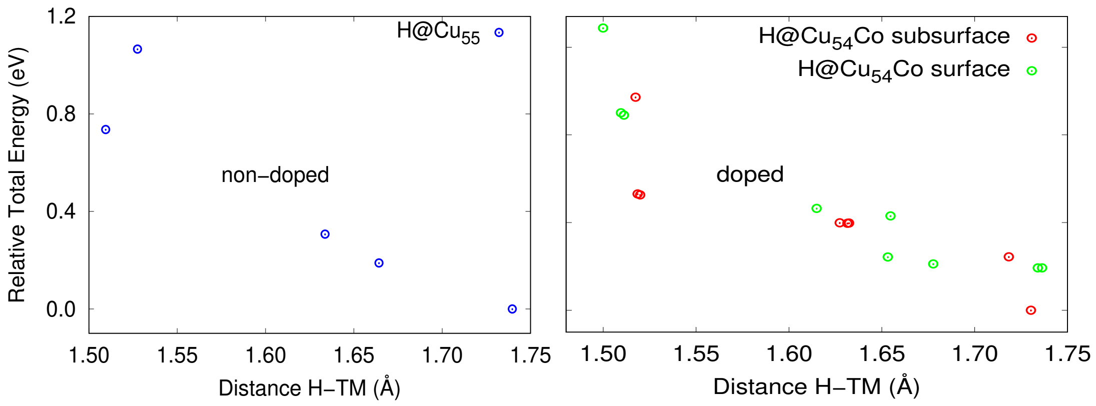

Figure S24: Relative energies of the adsorbed configurations of an H atom over the non-doped and doped clusters as a function of the shortest TM−H distance.

4.2.2 OH Adsorption

For the OH adsorption according to Figure S25, hollow configurations are the most stable when the cluster is not doped and when the dopant is in the surface. However, when the cobalt atom is in the subsurface, the bridge configuration is the most stable.

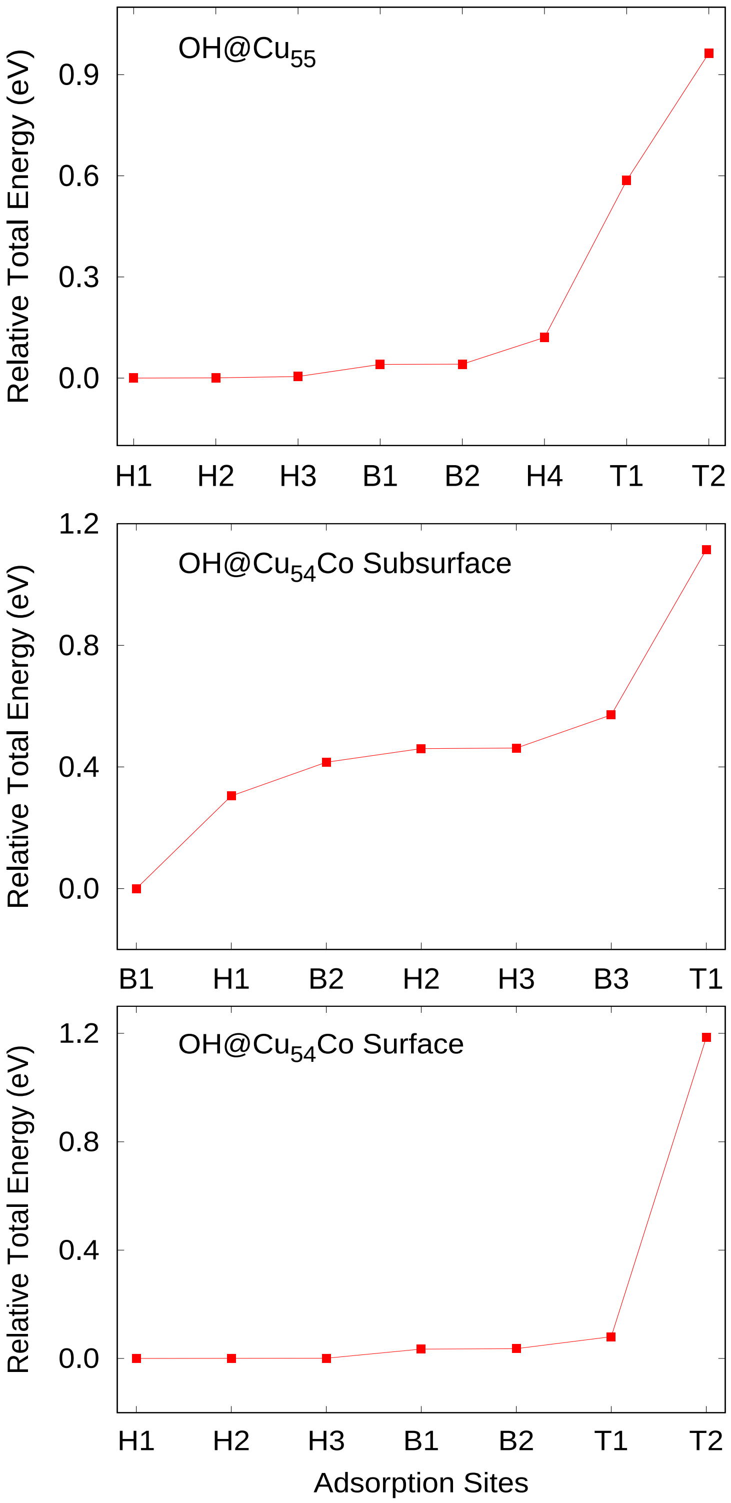

Figure S25: Relative energies of the optimized OH adsorption configurations on the Cu_{55} and $Cu_{54}Co$ clusters. The most energetically stable configuration is used as a reference.

Figure S26, S27 and S28 show the adsorption sites, geometries, and relative energies after optimization for the adsorption of OH. .

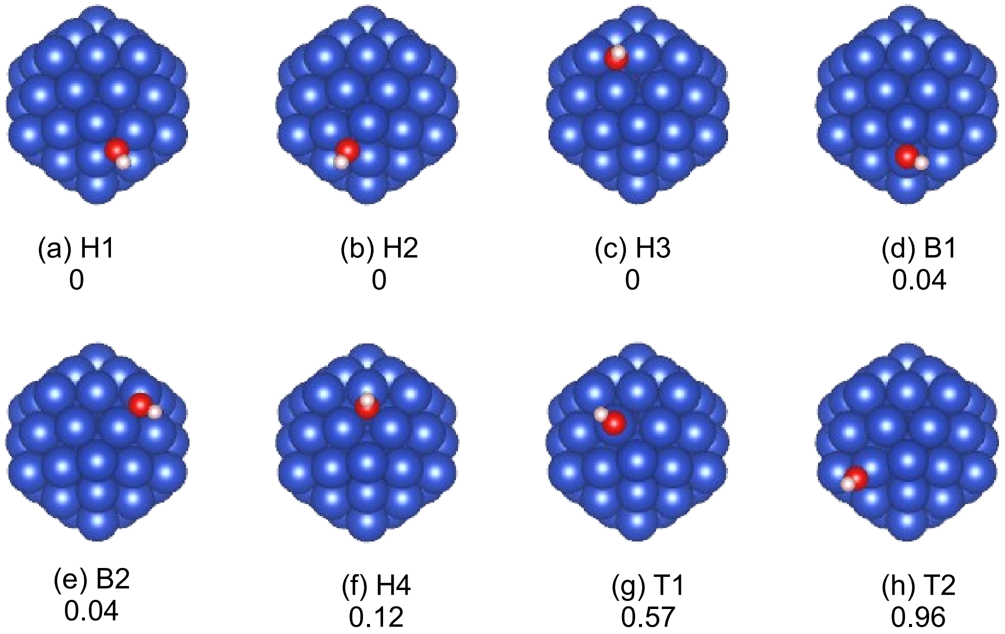

Figure S26: Optimized OH@Cu_{55} configurations. Relative energies are given in eV.

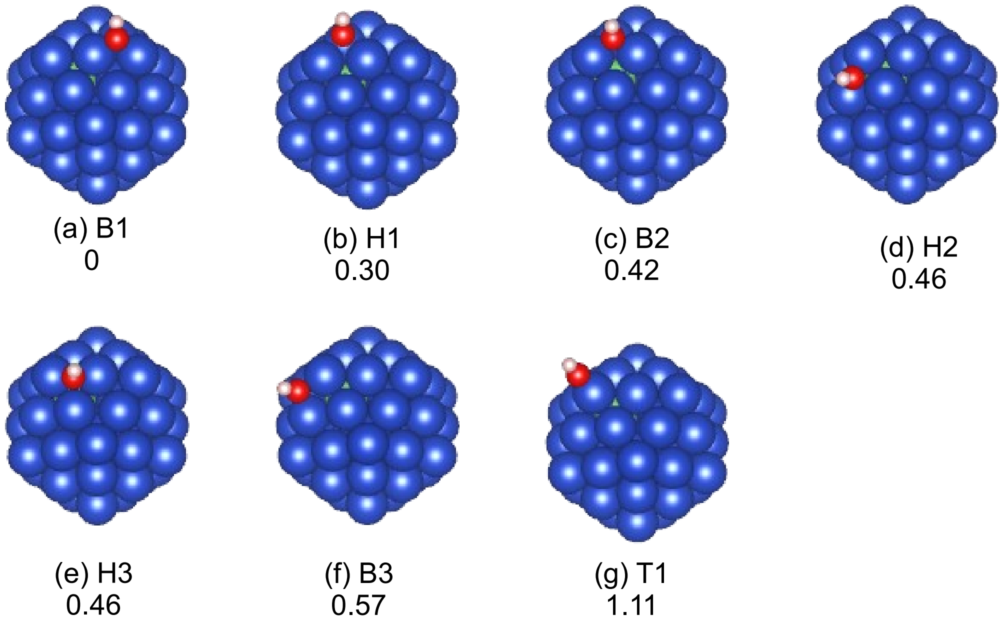

Figure S27: Optimized OH@$Cu_{54}Co$ configurations with the dopant in the subsurface position. Relative energies are given in eV.

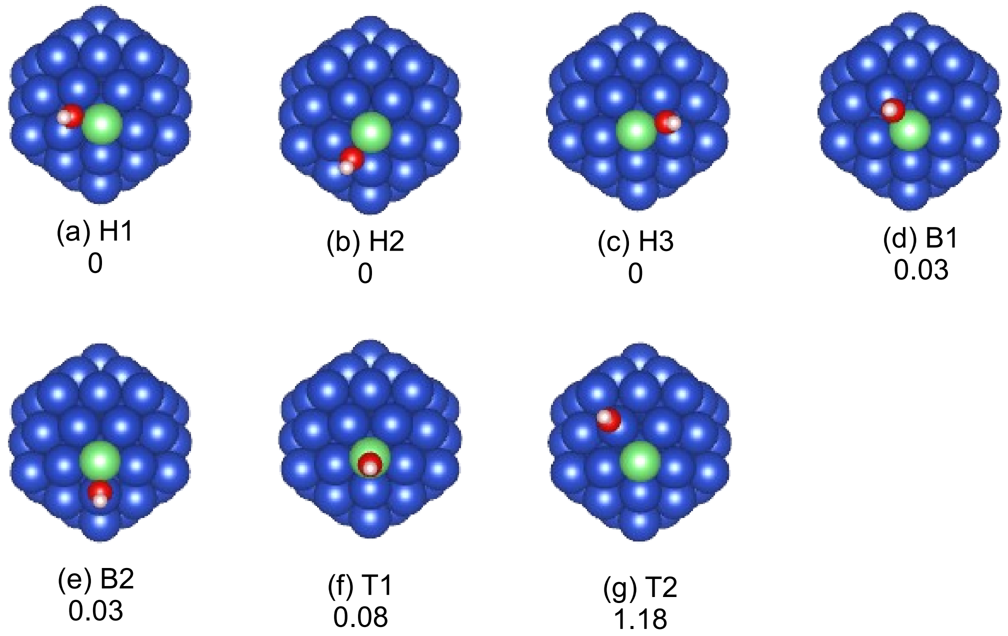

Figure S28: Optimized OH@$Cu_{54}Co$ configurations with the dopant in the surface position. Relative energies are given in eV.

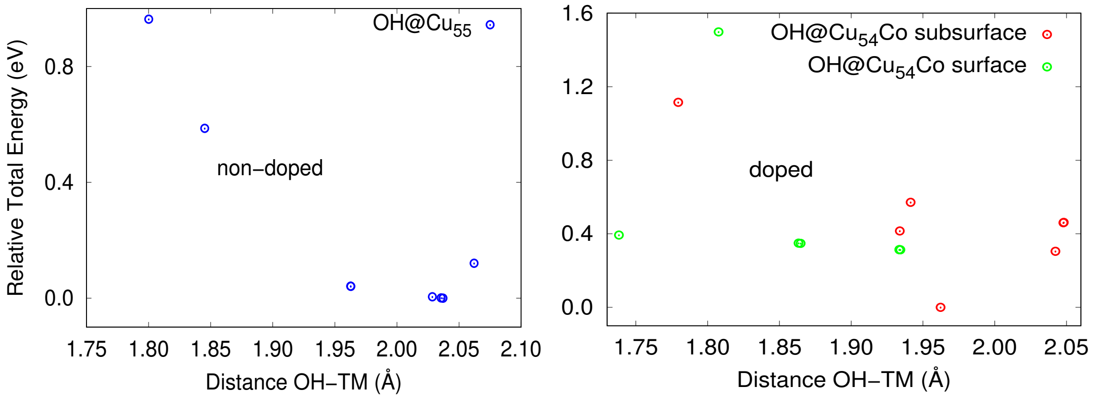

Figure S29: Relative energies of the adsorbed configurations of an OH molecule over the non-doped and doped clusters as a function of the shortest TM – O distance.

4.2.3 CO Adsorption

For the CO adsorption, according to Figure S30, when the cluster is non-doped, the most stable configurations are hollow. However, when the cluster is doped, all optimizations led to top configurations, even those that started on bridge or hollow configurations. It can be seen that the presence of the cobalt atom, even in the subsurface, drastically changes the site preferences.

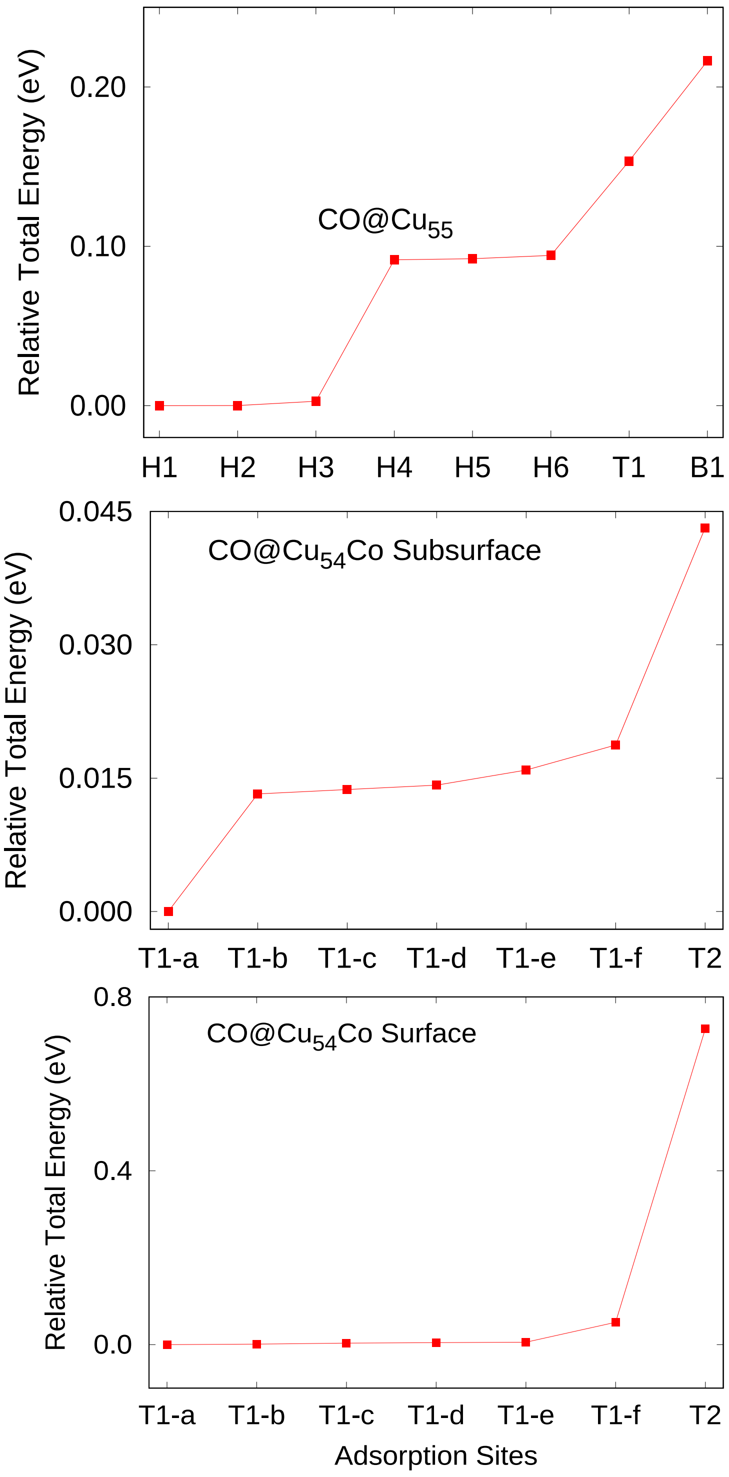

Figure S30: Relative energies of the optimized CO adsorption configurations on the Cu_{55} and $Cu_{54}Co$ clusters. The most energetically stable configuration is used as a reference.

Figures S31, S32 and S33 show the adsorption sites, geometries, and relative energy after optimization for the adsorption of CO.

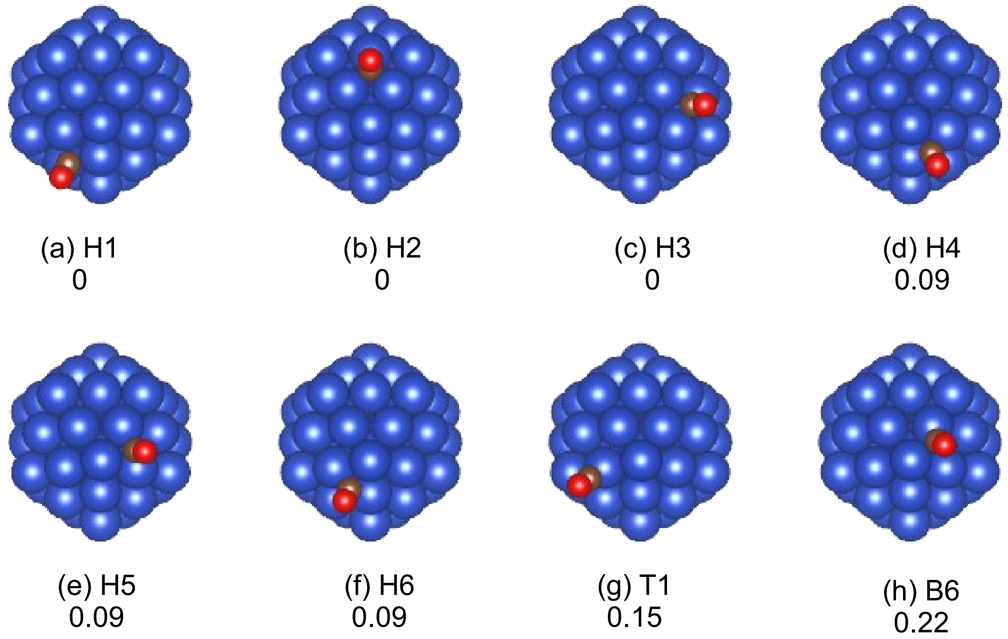

Figure S31: Optimized $CO@Cu_{55}$ configurations. Relative energies are given in eV.

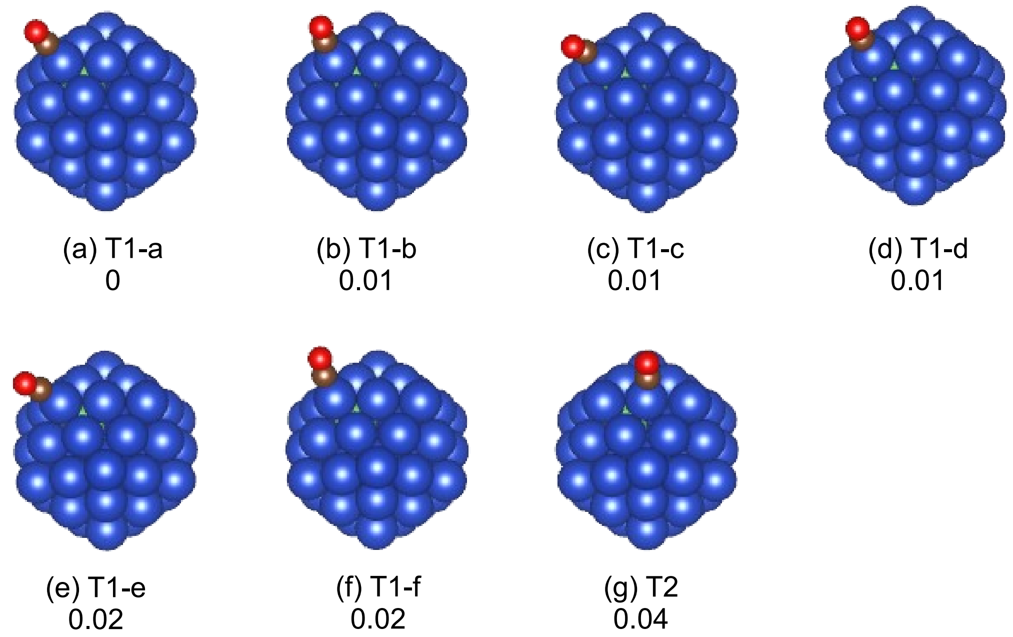

Figure S32: Optimized $CO@Cu_{54}Co$ configurations with the dopant in the subsurface position. Relative energies are given in eV.

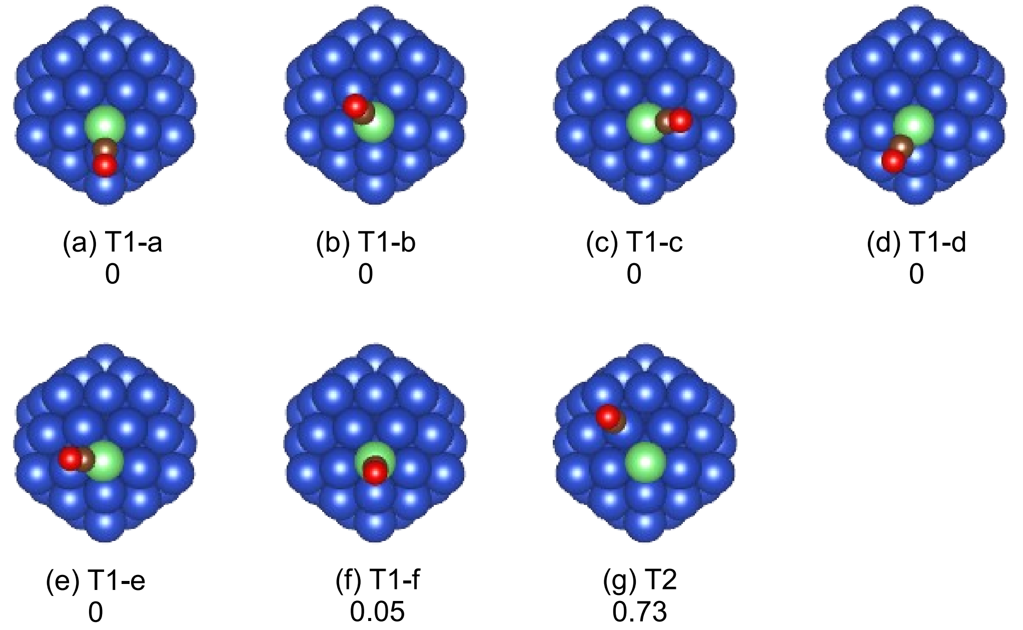

Figure S33: Optimized CO@Cu_{54}Co configurations with the dopant in the surface position. Relative energies are given in eV.

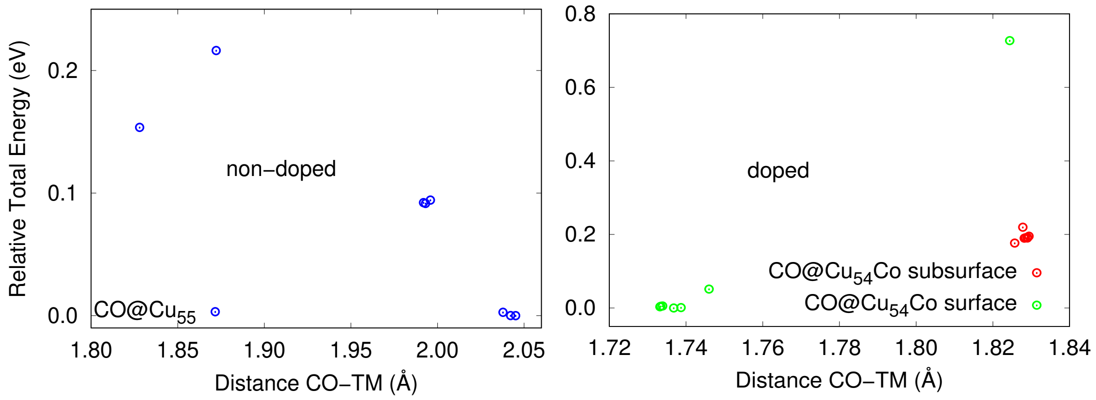

Figure S34: Relative energies of the adsorbed configurations of an CO molecule over the non-doped and doped clusters as afunction of the shortest TM−C distance.

4.2.4 COH Adsorption

As seen in Figure S35, the COH adsorption favors hollow configurations in all substrates.

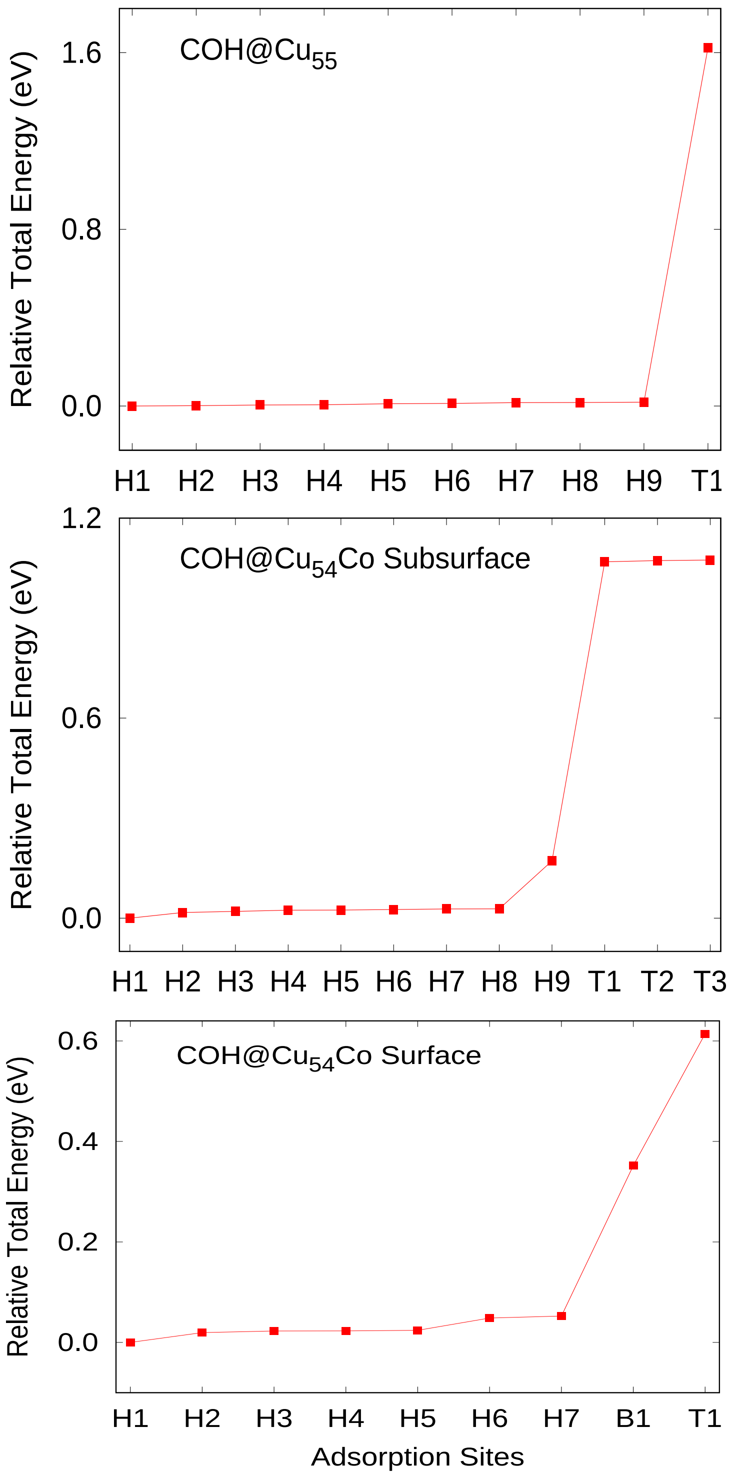

Figure S35: Relative energies of the optimized COH adsorption configurations on the Cu_{55} and $Cu_{54}Co$ clusters.

Figures S36, S37 and S38 show the adsorption sites, geometries and relative energies after optimization for the adsorption of COH.

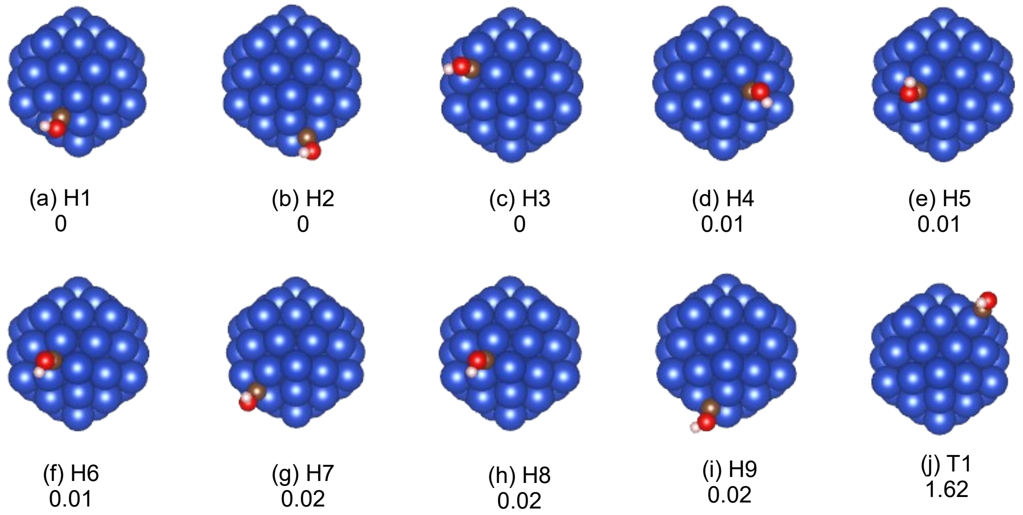

Figure S36: Optimized COH@Cu_{55} configurations. Relative energies are given in eV.

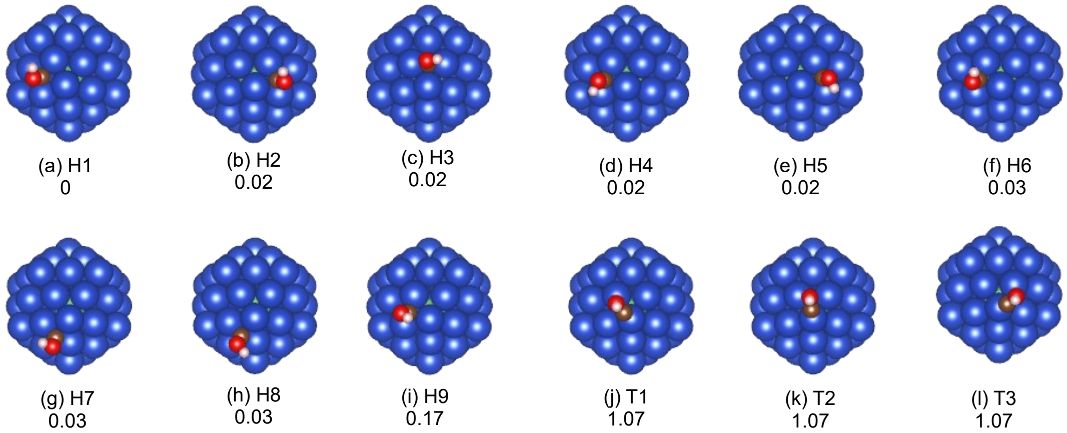

Figure S37: Optimized COH@Cu_{54}Co configurations with the dopant in the subsurface position. Relative energies are given in eV.

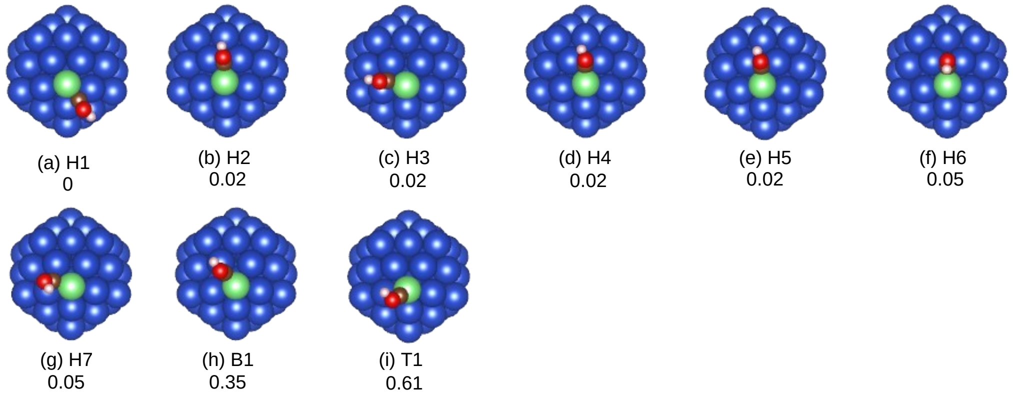

Figure S38: Optimized COH@Cu_{54}Co configurations with the dopant in the surface position. Relative energies are given in eV.

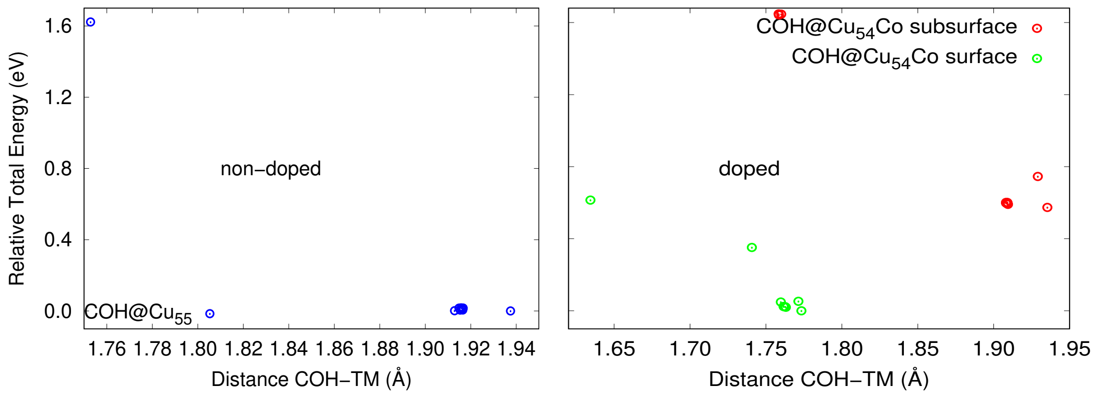

Figure S39: Relative energies of COH adsorbed on the non-doped and doped clusters as a function of the shortest TM – C distance.

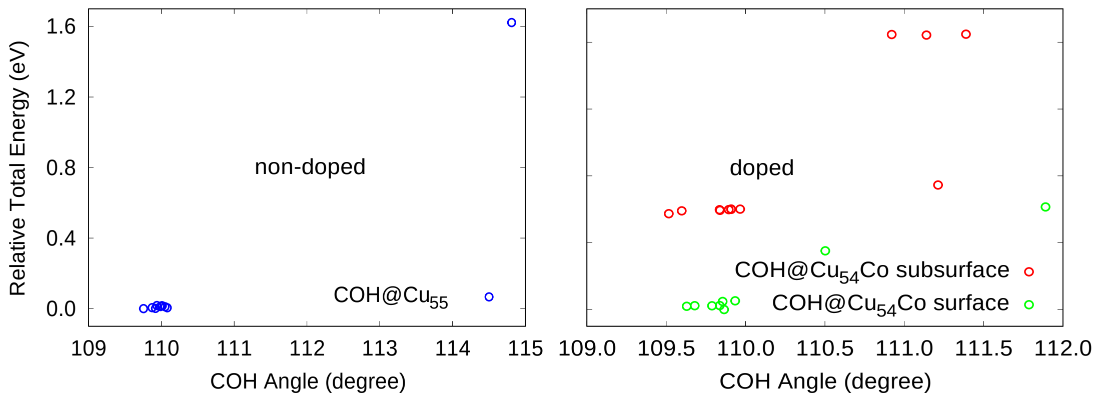

Figure S40: Relative energies of COH adsorbed on the non-doped and doped clusters as a function of the angle formed by C, O and H atoms. Using the same calculations parameters, the angle formed by C, O and H atoms in the isolated (gas-phase) molecule is 115.5 degrees.

4.2.5 HCO Adsorption

Figure S41 shows the relative energies for the various HCO adsorption configurations. The most stable structures are predicted to be of the bridge type, for both non-doped and doped cases.

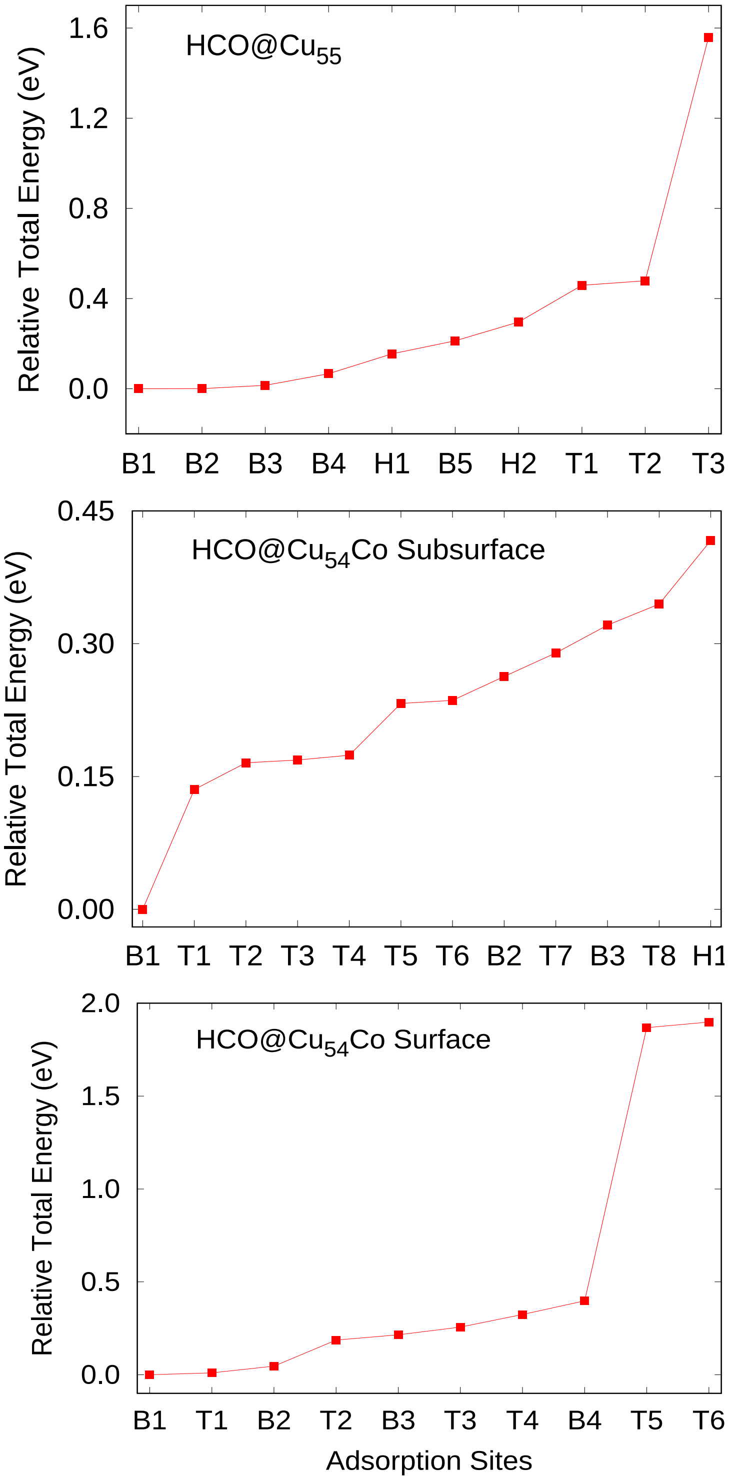

Figure S41: Relative energies of the optimized HCO adsorption configurations on the Cu_{55} and $Cu_{54}Co$ clusters.

Figures S42, S43 and S44 show the adsorption sites, geometries, and relative energies after optimization for the adsorption of HCO.

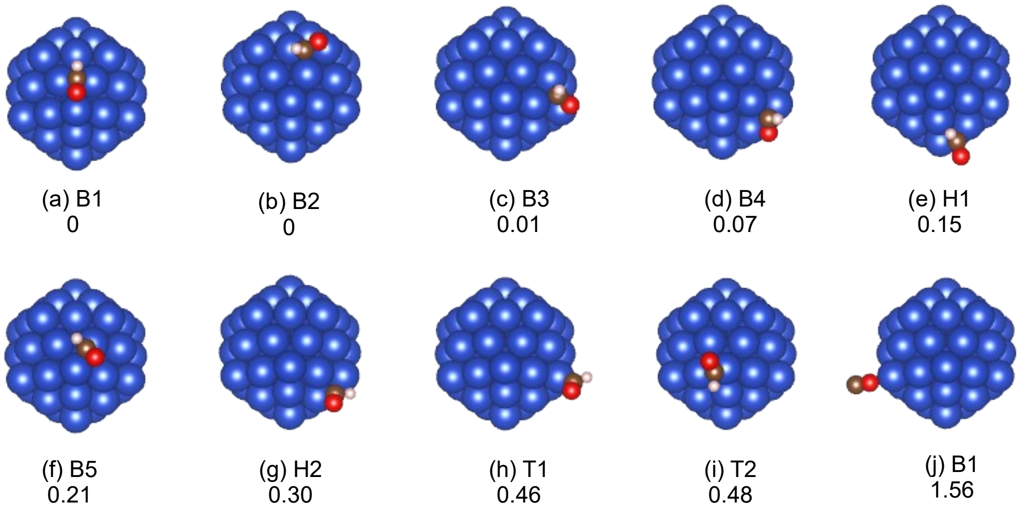

Figure S42: Optimized HCO@Cu_{55} configurations. Relative energies are given in eV.

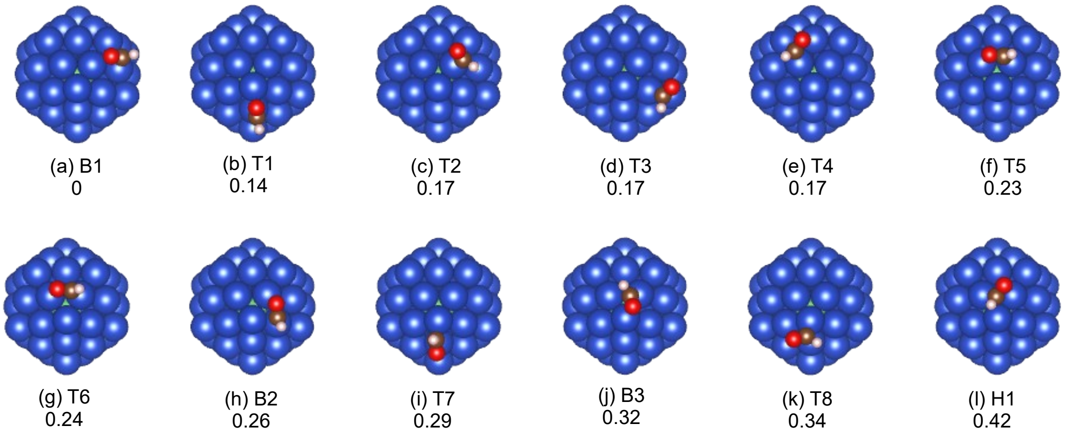

Figure S43: Optimized HCO@Cu_{54}Co configurations with the dopant in the subsurface position. Relative energies are given in eV.

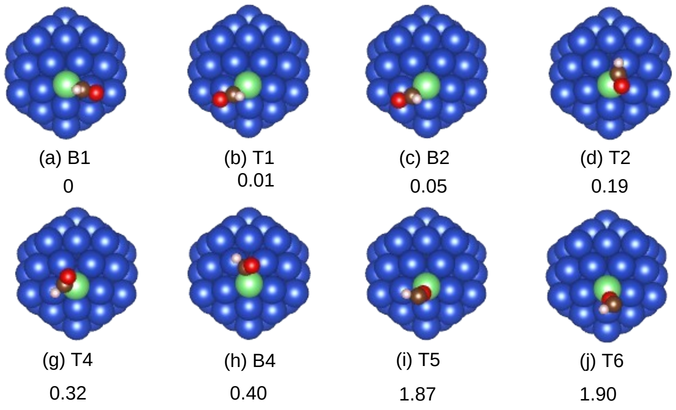

Figure S44: Optimized HCO@Cu_{54}Co configurations with the dopant in the surface position. Relative energies are given in eV.

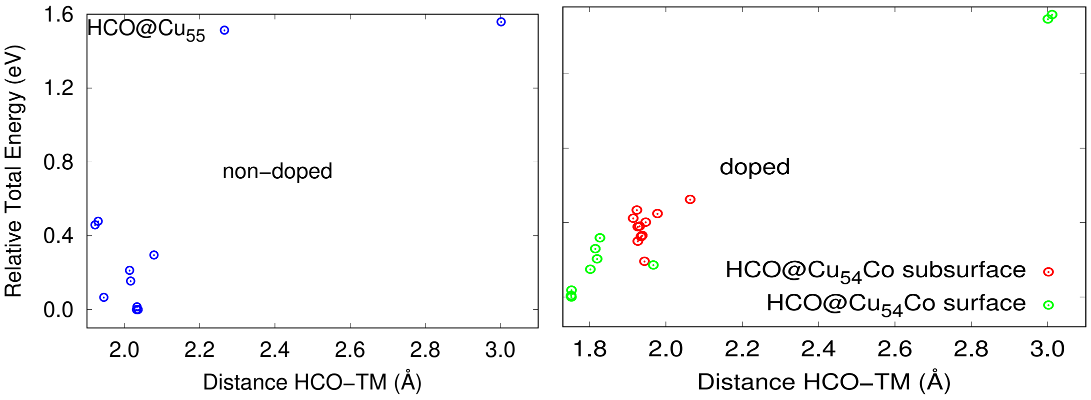

Figure S45: Relative energies of HCO adsorbed on the non-doped and doped clusters as function of the shortest Cu – C distance.

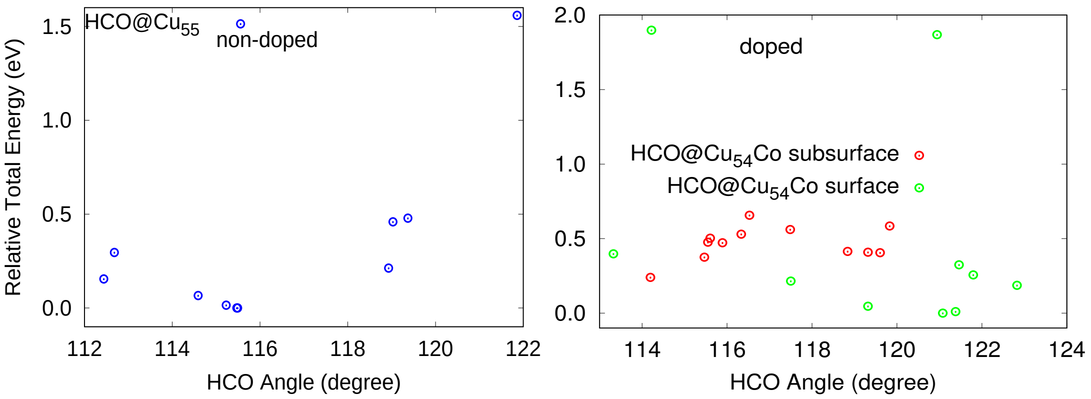

Figure S46: Relative energies of HCO adsorbed on the non-doped and doped clusters as function as a function of the angle formed by H, C and O atoms. In the gas phase, using the same calculation parameters the angle formed by C, O and H atoms are 124.18 degrees.

5 Energetic Contributions from Vibrational Calculations

As discussed in the main manuscript, we employ the computational hydrogen electrode (CHE) model[6,7] to evaluate free energy changes for the electrochemical steps. The contributions to the Gibbs free energy of the gas-phase species are given in Table S2, which took into account the ideal gas limit, following the approach described by Peterson et al.. An additional −0.51 eV is added as a gas phase corrections (GPC) for CO due to inconsistencies of thermochemical data calculated with the PBE functional.

Table S2: Values of ZPE, $\int C_p dT$, TS and the sum of these quantities, for the gas phase molecules.

Molecule	ZPE	$\int C_p dT$	TS	Total	GPC
H_2	0.278	0.091	0.433	−0.064	
OH	0.223	0.091	0.551	−0.237	
CO	0.132	0.091	0.686	−0.463	−0.51
COH	0.328	0.105	0.694	−0.261	
HCO	0.343	0.105	0.694	−0.246	
H_2O	0.564	0.104	0.67	−0.02	

In tables S3, S4, and S5 we present the contributions to the Gibbs free energy for adsorbed configurations on the unary, subsurface doped, and surface doped clusters, respectively.

Table S3: Values of ZPE, $\int C_p dT$, TS and the sum of these quantities, for the molecules adsorbed in unary clusters. E_{sol} obtained from the literature, SC1[7] and SC2.[8].

System	ZPE	$\int C_p dT$	TS	Total	E_{sol}^{SC1}	E_{sol}^{SC2}
H@Cu_{55}	0.17	0.004	0.005	0.179	0.00	0.00
OH@Cu_{55}	0.342	0.05	0.082	0.474	−0.50	−0.58
CO@Cu_{55}	0.181	0.072	0.132	0.385	−0.10	0.00
COH@Cu_{55}	0.472	0.075	0.131	0.678	−0.25	−0.45
HCO@Cu_{55}	0.454	0.074	0.132	0.66	−0.10	0.00

Table S4: Values of ZPE, $\int C_p dT$, TS and the sum of these quantities, for the molecules adsorbed in unary clusters doped in the subsurface. E_{sol} obtained from the literature, SC1[7] and SC2.[8].

System	ZPE	$\int C_p dT$	TS	Total	E_{sol}^{SC1}	E_{sol}^{SC2}
H@Cu_{54}Co	0.164	0.005	0.006	0.175	0.00	0.00
OH@Cu_{54}Co	0.351	0.049	0.084	0.484	−0.50	−0.58
CO@Cu_{54}Co	0.189	0.055	0.109	0.353	−0.10	0.00
COH@Cu_{54}Co	0.47	0.076	0.136	0.682	−0.25	−0.45
HCO@Cu_{54}Co	0.461	0.071	0.127	0.659	−0.10	0.00

Table S5: Values of ZPE, $\int C_p dT$, TS and the sum of this quantities, for the molecules adsorbed in unary clusters doped in the surface. E_{sol} obtained from the literature, SC1[7] and SC2.[8].

System	ZPE	$\int C_p dT$	TS	Total	E_{sol}^{SC1}	E_{sol}^{SC2}
H@Cu_{54}Co	0.216	0.005	0.006	0.227	0.00	0.00
OH@Cu_{54}Co	0.342	0.05	0.083	0.475	−0.50	−0.58
CO@Cu_{54}Co	0.198	0.071	0.147	0.416	−0.10	0.00
COH@Cu_{54}Co	0.474	0.078	0.14	0.692	−0.25	−0.45
HCO@Cu_{54}Co	0.42	0.064	0.126	0.61	−0.10	0.00

References

1 Perdew, J. P.; Burke, K.; Ernzerhof, M. Generalized Gradient Approximation Made Simple. *Phys. Rev. Lett.* **1996**, *77*, 3865–3868, DOI: 10.1103/physrevlett.77.3865.

2 Da Silva, J. L. F. Effective Coordination Concept Applied for Phase Change $(GeTe)_m(Sb_2Te_3)_n$ Compounds. *J. Appl. Phys.* **2011**, *109*, 023502, DOI: 10.1063/1.3533422.

3 Da Silva, J. L. F.; Kim, H. G.; Piotrowski, M. J.; Prieto, M. J.; Tremiliosi-Filho, G. Reconstruction of Core and Surface Nanoparticles: The Example of Pt_{55} and Au_{55}. *Phys. Rev. B* **2010**, *82*, 205424, DOI: 10.1103/physrevb.82.205424.

4 Piotrowski, M. J.; Ungureanu, C. G.; Tereshchuk, P.; Batista, K. E. A.; Chaves, A. S.; Guedes-Sobrinho, D.; Da Silva, J. L. F. Theoretical Study of the Structural, Energetic, and Electronic Properties of 55-atom Metal Nanoclusters: A DFT Investigation Within van der Waals Corrections, Spin-Orbit Coupling, and PBE+U of 42 Metal Systems. *J. Phys. Chem. C* **2016**, *120*, 28844–28856, DOI: 10.1021/acs.jpcc.6b10404.

5 Grimme, S.; Antony, J.; Ehrlich, S.; Krieg, H. A Consistent and Accurate *Ab initio* Parametrization of Density Functional Dispersion Correction (DFT-D) for the 94 Elements H-Pu. *J. Chem. Phys.* **2010**, *132*, 154104, DOI: 10.1063/1.3382344.

6 Nørskov, J. K.; Rossmeisl, J.; Logadottir, A.; Lindqvist, L.; Kitchin, J. R.; Bligaard, T.; Jónsson, H. Origin of the Overpotential for Oxygen Reduction at a Fuel-Cell Cathode. *J. Phys. Chem. B* **2004**, *108*, 17886–17892.

7 Peterson, A. A.; Abild-Pedersen, F.; Studt, F.; Rossmeisl, J.; Nørskov, J. K. How copper catalyzes the electroreduction of carbon dioxide into hydrocarbon fuels. *Energy Environ. Sci.* **2010**, *3*, 1311–1315, DOI: 10.1039/C0EE00071J.

8 Rendón-Calle, A.; Builes, S.; Calle-Vallejo, F. Substantial improvement of electrocatalytic predictions by systematic assessment of solvent effects on adsorption energies. *Applied Catalysis B: Environmental* **2020**, *276*, 119147, DOI: 10.1016/j.apcatb.2020.119147.
